# Gegen Qinlian decoction alleviates DSS-induced colitis in mice through coordinated modulation of gut microbiota, serum metabolome, and colonic γδT cell responses

**DOI:** 10.3389/fimmu.2026.1765637

**Published:** 2026-04-02

**Authors:** Yanhua Shao, Dan Wang, Yijun Zhang, Jiahao Zhai, Enhui Wu, Lanfang Tan, Xiangliang Deng, Fengyun Wang, Yunfei Liang, Minghua Xian, Qingfei Xian

**Affiliations:** 1 School of Chinese Materia Medica, Guangdong Pharmaceutical University, Guangzhou, Guangdong, China; 2 Guangxi Key Laboratory of Functional Phytochemicals Research and Utilization, Guangxi Institute of Botany, Chinese Academy of Sciences, Guilin, China; 3 School of Traditional Chinese Medicine, Guangdong Pharmaceutical University, Guangzhou, Guangdong, China; 4 Guangxi Engineering Research Center of Innovative Preparations for Natural Medicine, Guangxi Wuzhou Pharmaceutical (Group) Co., Ltd, Wuzhou, China; 5 Department of Neurosurgery, First School of Clinical Medicine, The First Affiliated Hospital of Guangdong Pharmaceutical University, Guangzhou, Guangdong, China

**Keywords:** Gegen Qinlian decoction, gut microbiota, immunomodulatory effects, serum metabolites, ulcerative colitis, γδτ cells

## Abstract

**Background:**

Ulcerative colitis (UC) is a chronic, relapsing inflammatory bowel disease. Despite advances in current therapies, safer, more effective drugs are urgently needed. Traditional Chinese herbal formula Gegen Qinlian Decoction (GQD) has been used for gastrointestinal disorders, including UC, though its exact mechanisms require further clarification.

**Objective:**

This study aimed to systematically evaluate the therapeutic effects of GQD in UC mice, focusing on serum metabolomics, gut microbiota, and immunomodulatory mechanisms.

**Methods:**

A dextran sulfate sodium (DSS)-induced mouse model of UC was established. Serum metabolomics and 16S rRNA sequencing analysis of GQD’s effects on metabolites and gut microbiota. Correlation analysis and network pharmacology identified potential targets and pathways of GQD. Immunofluorescence detected the expression of γδT cells, TNF-α, IFN-γ, and IL-17 proteins in the colonic tissue.

**Results:**

Using UPLC-QE-Orbitrap-MS, 71 compounds were identified in the GQD quality control analysis. GQD markedly attenuated colonic histopathological damage and suppressed serum pro-inflammatory cytokines IFN-γ, IL-17, and TNF-α. It also modulated key serum metabolites, including succinic acid, glyoxylate, and xanthine, which are primarily involved in amino acid and purine metabolic pathways. GQD further influenced intestinal microbial diversity and composition. Joint analysis revealed GQD modulates gut microbiota, serum amino acid and purine metabolism, and inflammation pathways. Immunohistochemical results demonstrated enhanced infiltration of γδT cells following GQD treatment, accompanied by reduced protein expression levels of TNF-α, IFN-γ, and IL-17.

**Conclusion:**

GQD exerts therapeutic effects on UC by reshaping gut microbiota composition and metabolic activities, thereby ameliorating intestinal mucosal injury, regulating γδT cell-mediated immune responses, and influencing amino acid and purine metabolic pathways.

## 1 Introduction

Ulcerative colitis (UC) is an inflammatory bowel disease characterized by chronic inflammation of the colonic and rectal mucosa (1). Its pathological process is frequently accompanied by disruption of the mucosal barrier, leading to increased intestinal epithelial permeability and loss of the mucus layer. This exposes the intestinal microbiota and its products to the immune cells of the lamina propria, triggering abnormal immune responses (2). Clinical manifestations include recurrent abdominal pain, diarrhea, mucus-blood stools, and weight loss. Severe cases may progress to intestinal perforation, toxic megacolon, or even malignancy (3). The global burden of ulcerative colitis has risen significantly (4, 5), with prevalence rates exceeding 0.3% in North America, Australia, and Europe. Future projections indicate that the incidence of inflammatory bowel disease worldwide will continue to climb (6). Current conventional therapies, including 5-aminosalicylic acid preparations, glucocorticoids, immunosuppressants, and biologics, face limitations such as incomplete efficacy, adverse reactions, and substantial economic burdens (7). There is an urgent need to develop novel therapeutic strategies targeting multiple pathogenic pathways.

The pathogenesis of UC is complex, involving the interplay of multiple factors, including genetics, environment, gut microbiota dysbiosis, host metabolic disorders, and immune dysfunction (8–10). Regarding genetic factors, genome-wide association studies (GWAS) have identified variants in genes such as PARK7, IL-23R, and GNA12 as being closely associated with susceptibility to UC. These genes predominantly participate in intestinal barrier function, immune responses, and autophagy processes (11). Among environmental factors, high-fat and low-fibre diets, smoking, psychological stress, and medication abuse (such as non-steroidal anti-inflammatory drugs) can trigger abnormal immune responses in the gut (12, 13). Gut microbiota dysbiosis plays a pivotal role in UC pathogenesis, characterised by reduced beneficial bacteria (e.g., Bifidobacterium, Lactobacillus) and increased pathogenic bacteria (e.g., Bacteroides fragilis, Escherichia coli). This imbalance diminishes short-chain fatty acid (SCFA) synthesis and compromises intestinal mucosal barrier function (14). Furthermore, immune dysregulation constitutes a core pathological mechanism in UC. Excessive T-cell activation triggers substantial release of pro-inflammatory cytokines (IFN-γ, IL-17, TNF-α, IL-6) while simultaneously impairing expression of anti-inflammatory factors (IL-10, TGF-β). This disruption of intestinal immune equilibrium further exacerbates the inflammatory response (15).

Gegen Qinlian Decoction (GQD) is a traditional Chinese medicinal formula clinically employed for treating ulcerative colitis and was first documented in the Treatise on Cold Damage Disorders (16). This formula comprises four herbs: *Puerariae Lobatae Radix* (Lobed Kudzuvine Root, Gegen), *Scutellariae Radix* (Baical Skullcap Root, Huangqin), *Coptidis Rhizoma* (Coptis Root, Huanglian), and *Glycyrrhizae Radix et Rhizoma* (liquorice root, Gancao) (16). Notably, multiple studies have independently investigated the single herbs Gegen, Huangqin, and Huanglian within the formula, or their primary active constituents, confirming their respective efficacy in treating colitis. The formula further concentrates these bioactive compounds (17–20), including flavonoids (e.g., puerarin, baicalin, baicalein), alkaloids (e.g., berberine, berberine hydrochloride), and polysaccharides (e.g., Pueraria lobata polysaccharide, glycyrrhiza polysaccharide). These components work synergistically through multiple targets to exert therapeutic effects, such as anti-inflammatory, antioxidant, immunomodulatory, and intestinal mucosal barrier-repairing activities (21, 22). GQD ameliorate experimental colitis through antioxidant, barrier-protective, metabolic, and microbiota-modulating effects (23, 24). However, these mechanisms have been investigated in isolation, leaving the crucial interplay among gut microbiota, host metabolism, and mucosal immunity, along with its central role in the therapeutic action of GQD, largely unexamined (25, 26).

**Figure 1:**
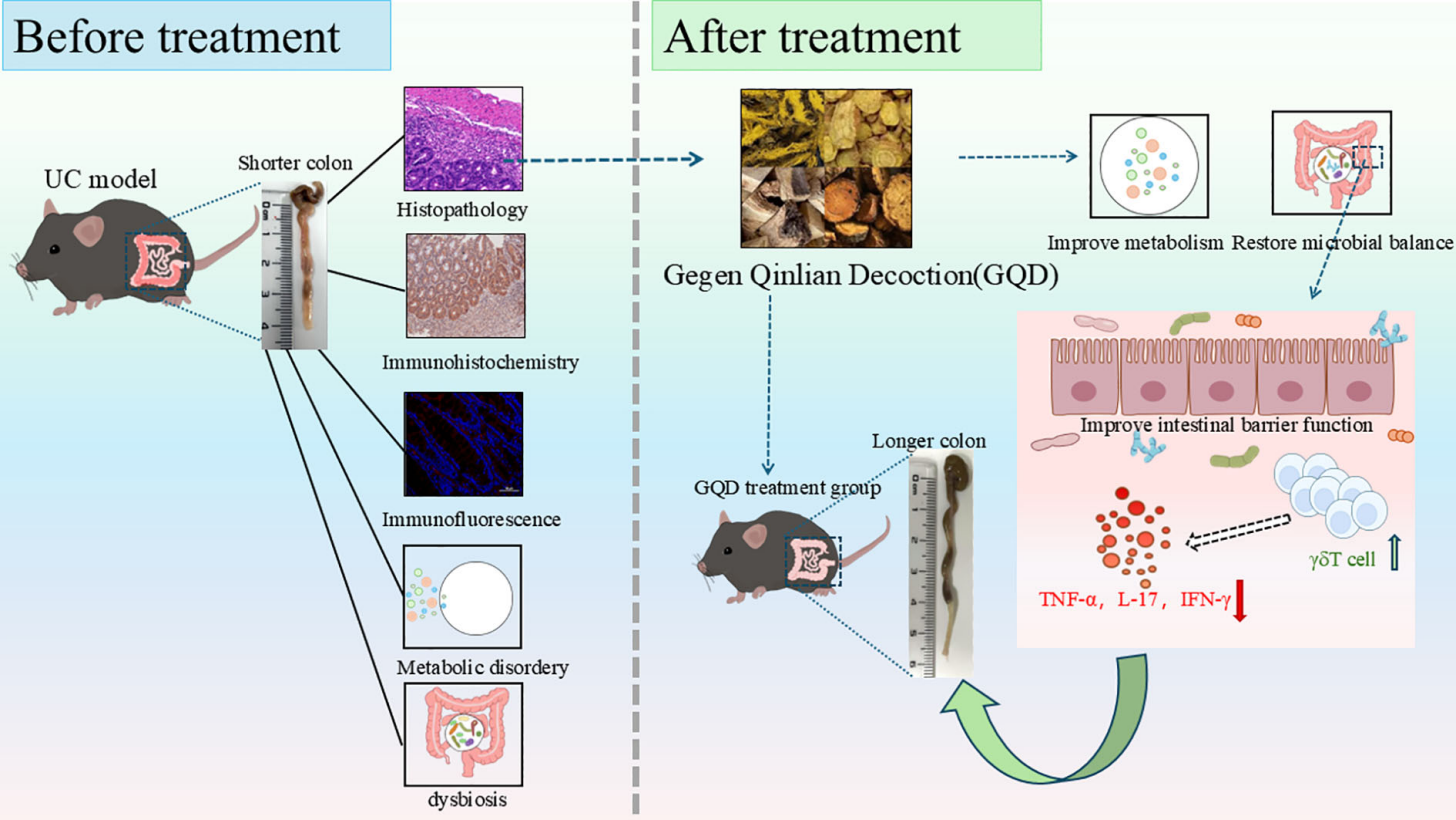
Schematic design of the study.

γδT cells are critical regulators of intestinal immunity, with IFN-γ and IL-17-producing subsets implicated in UC pathogenesis and tissue-resident subsets essential for mucosal repair (27–30). However, whether GQD modulates this key innate immune compartment remains completely unknown. To address this knowledge gap, we employed a DSS-induced colitis model to investigate GQD’s therapeutic mechanism, focusing on the interplay between gut microbiota, host metabolism, and colonic γδT cells. Using multi-omics integration and immunophenotyping, we demonstrate that GQD exerts a pivotal role via the gut microbiota, simultaneously influencing serum metabolic alterations and the functional regulation of colonic γδT cells, thereby alleviating UC in mice, providing novel mechanistic insight into its clinical application for UC. (**Figure 1**).

## 2 Material and methods

### 2.1 Experimental animal and feeds

This experiment utilised male 6-8 week-old SPF-grade C57BL/6 mice, weighing 20-22g, procured from Guangzhou Yancheng Biotechnology Co., Ltd. Following approval by the Ethics Committee of Guangdong Pharmaceutical University, the mice were housed in the SPF animal facility at the university’s Animal Centre. The laboratory environment maintained a temperature of approximately 24.0 ± 2.0°C and relative humidity of approximately 50 ± 10%. A 12-hour light-dark cycle was employed, with feed and water provided regularly. Experiments commenced after a 7-day acclimatisation period. Ethics Approval Number: gdpulac2023130.

### 2.2 Chemicals and reagents

*Puerariae Lobatae Radix* (Gegen, #210501), *Scutellariae Radix* (Huangqin, #220501), *Coptidis Rhizoma* (Huanglian, #220301), and *Glycyrrhizae Praeparata cum Melle Radix et Rhizoma* (Zhigancao, #211101) were purchased from Shizhen Pharmaceutical (Guangdong). Sodium dextran sulfate (DSS, #MB5535) and mesalazine (5-ASA, #MB7539) were obtained from Meilun Biotechnology Co., Ltd. TCR γ/δ (UC7-13D5) (#sc-19608) was purchased from Santa Cruz Biotechnology. The enzyme-linked immunosorbent assay (ELISA) based kits of IL-17 (#MM-0170M1), TNF-α (#MM-0132M1) and IFN-γ (#MM-0182M1) assay kits were procured from Jiangsu Meimian Industrial Co., Ltd. TransStart Fastpfu DNA Polymerase (#AP221-02) was sourced from TransGen Biotech (Beijing). The TruSeqTM DNA Sample Preparation Kit was purchased from Illumina. The AxyPrep DNA Gel Extraction Kit (#FC-121-2002) was obtained from AXYGEN in the United States. The DNA extraction kit (#D5625-01) was sourced from Omega in the United States. Anhydrous ethanol (#100092683), xylene (#10023418) and neutral gum (#10004160) were all procured from China National Medicines Corporation Ltd.; HE staining kit (# G1003) and PBS buffer (#G0002) were procured from Wuhan Servicebio Technology Co., Ltd; Sodium citrate antigen retrieval solution (#20220308) was procured from Beijing Solarbio Science & Technology Co., Ltd; BSA (#20180218) and 4% paraformaldehyde (#70085400) were both purchased from Beijing Langjieko Technology Co., Ltd.

### 2.3 Preparation of GQD and UPLC-QE-Orbitrap-MS quality control analysis

Take 15 g of Gegen, 9 g of Huangqin, 9 g of Huanglian, and 6 g of Zhigancao. The mixture was soaked in eightfold water for 30 min, brought to a boil over high heat, reduced to a gentle simmer (approximately 100°C), and decocted for 15 min. The residue was filtered, and eightfold the volume of water was added to it. The mixture was decocted a second time using the same method, filtered, and the two filtrates were combined and concentrated to a solution with a drug concentration of 1 g/mL (i.e., 1 g of crude drug per mL). The solution was diluted with distilled water when required.

Qualitative analysis of non-concentrated GQD was performed using UPLC-QE-Orbitrap-MS, with the following chromatographic conditions: an ACQUITY UPLC^®^ BEH C18 column (2.1×100 mm, 1.7 μm) was used, with an injection volume of 1 μL and a column temperature of 35°C. Mobile phase A consists of 0.1% formic acid in water, and mobile phase B comprises acetonitrile. Gradient elution conditions are as follows: 0~2 min, 5% B; 2~16 min, 50% B; 16~22 min, 60% B; 22~32 min, 90% B; 32~33 min, 90% B; 33~34 min, 5% B; 34~35 min, 5% B. The flow rate was set to 0.3 mL/min. Mass spectrometry conditions: electrospray ionization (ESI) source, positive and negative ion scanning, capillary voltage +3.5 kV (Positive), -3.2 kV (Negative); Sheath gas flow rate: 45 arb; Aus gas flow rate: 10 arb; Capillary temperature: 300°C; Aus gas heater temperature: 300°C; Tandem mass spectrometry primary scan range: m/z 100-1500, resolution 70000; Secondary mass spectrometry employs dynamic data-dependent scanning, resolution 17500, collision energies 10, 25, 40 eV.

### 2.4 Mouse model of ulcerative colitis

Following one week of adaptive feeding, mice were randomly assigned to six groups (n=8 per group): control group (Con), DSS group (DSS), mesalazine treatment group (5-ASA, 200 mg/kg) (31, 32), low-dose GQD treatment group (GQD-L, 5 g/kg) and Medium-dose GQD treatment group (GQD-M, 10 g/kg), High-dose GQD treatment group (GQD-H, 20 g/kg). The GQD stock solution was diluted to 0.25, 0.50, and 1.0 g/mL for the low−, medium−, and high−dose groups, respectively. This is based on clinical human dose estimates. The Con group received free access to clean water, whereas the other groups were administered a 3% DSS solution (33), which was switched to clean water after seven days. Concurrently, the Con and DSS groups received 0.9% saline solution via gastric lavage, and all treatment groups were administered the corresponding drugs at the prescribed dosage for 10 days (34). All mice were orally administered a volume of 0.2 mL/10 g body weight per day. This regimen was maintained for 10 consecutive days and administered once daily. The daily disease activity indices were recorded. On day 11, the mice were anesthetized with sodium pentobarbital. Blood was collected by orbital venipuncture, and serum was separated and stored at -20°C. Colon and spleen tissues were harvested and preserved in 4% paraformaldehyde.

### 2.5 Colitis assessment

Throughout the experimental period, daily observations and recordings were made of all mice’s body weight, faecal consistency, and faecal bleeding status. Based on scores for weight loss, faecal consistency, and faecal blood, the disease activity index (DAI) was calculated as the mean score of these three parameters. The assessment criteria have been slightly adjusted with reference to the literature (31); the assessment criteria are set out in **Supplementary Table 1**. Following the conclusion of the treatment period, mice were euthanised by cervical dislocation under anaesthesia. Organs, including the spleen and colon, were excised, rinsed thoroughly with PBS, and the colon length was measured using a ruler. The spleen was weighed using an analytical balance to calculate the spleen index. Spleen index (mg/g) = spleen weight (mg)/body weight (g), (n=6).

### 2.6 Serum inflammatory cytokine level testing

Blood samples were collected and allowed to stand at room temperature for 30 min, then centrifuged at 3000 rpm for 10 min at 4°C. The supernatant was collected to the obtain serum. Following the provided protocol, the MEIMIAN mouse assay kit was used to detect the levels of the inflammatory cytokines IFN-γ, IL-17, and TNF-α in the serum, (n=6).

### 2.7 Histological analysis

Colon tissue was fixed in 4% paraformaldehyde for 24 h, followed by paraffin embedding. Sections 4 μm thick were prepared for HE staining (35). Under microscopic examination, the degree of colonic injury and inflammatory cell infiltration was assessed based on the results of microscopic imaging. Histological scoring was performed with minor modifications based on previously reported methods (36), focusing on three independent parameters: (a) ulceration (score 0-4: none, one focus, two foci, three to four foci, extensive), (b) epithelial cell changes (score 0-4: normal, goblet cell loss, extensive goblet cell loss, crypt absence, extensive crypt loss), and (c) inflammatory infiltration (score 0-4: none, peri-cryptal, reaching the muscularis mucosa, widespread in muscularis mucosa with thickening, submucosal infiltration). The total score for each sample was then calculated as the average of the scores from these three parameters, (n=6).

### 2.8 Serum metabolomics analysis

#### 2.8.1 Sample preparation

Take 100 microliters of serum and add 1000 microliters of extraction solution (methanol: acetonitrile: water = 2:2:1, volume ratio). Mix the solution evenly using vortex agitation. Perform ultrasonic treatment in iced water for 10 min, followed by liquid nitrogen treatment for 1 min. Repeat this process three times. Allow the sample to stand at -20°C for 1 h. Centrifuge the sample at 4°C and 3000 rpm for 15 min. Collect the supernatant and dry it using a nitrogen evaporator. Redissolve the dried sample with 100 microliters of 50% acetonitrile-water solution (acetonitrile: water = 1:1, volume ratio). Vortex the solution for 30 s and perform ultrasonic treatment in iced water for 10 min. Centrifuge the solution again at 4°C and 3000 rpm for 15 min. Collect the supernatant for further analysis. (n=3).

#### 2.8.2 UPLC-Q-TOF/MS detection conditions

For UPLC-Q-TOF/MS detection, the chromatographic conditions were set as follows: a UPLC BEH Amide column (2.1×100 mm, 1.7 μm) was used, with an injection volume of 5 μL and a column temperature of 55°C. The mobile phase A consisted of 100% water with 25 mM CH_3_COONH_4_ and 25 mM NH_3_·H_2_O, while the mobile phase B was 100% acetonitrile. The gradient elution conditions were as follows: 0~1 min, 85% B; 1~12 min, 65% B; 12~12.1 min, 40% B; 12.1~15 min, 40% B; 15~15.1 min, 85% B; 15.1~20 min, 85% B. The flow rate was set at 0.3 mL/min.

In mass spectrometry, the ionization method was an ESI source, with an ion source temperature of 600°C and an ion source voltage of either -4500 V or 5500 V. The curtain gas was set at 20 psi, while the nebulizer gas and auxiliary gas were both set at 60 psi.

#### 2.8.3 Data analysis

The raw data was converted into mzXML format, and peak alignment, RT correction, M/Z mass comparison, and peak area extraction were performed using XCMS software.

The structures of metabolites were accurately matched using both first-order and second-order spectra (<25 ppm). This matching was done based on our established database (VGDB) as well as public databases such as METLIN, MassBank, mzCloud, HMDB, KEGG, MetaCyc, Lipidmaps, and MS-Dial.

The data were standardized using SIMCA14.1 and MetaboAnalyst 5.0 databases. Statistical analysis and graphical representation of the data were also conducted.

### 2.9 Detection of gut microbiota

#### 2.9.1 Sample collection

Following the final drug administration, fecal samples were obtained from mice using the tail-lift collection technique. Sterile tweezers, disinfected with alcohol, were employed to transfer the feces into sterile Eppendorf tubes. After each sample collection, the tweezers were sterilized with alcohol prior to proceeding with the next mouse. All collected fecal specimens were promptly stored at -80°C for preservation, (n=3).

#### 2.9.2 16S rRNA detection

The sequencing process involves the extraction of DNA from environmental samples, the design and synthesis of primer adapters, PCR amplification and purification of products, quantification and normalization of PCR products, construction of PE libraries, and Illumina sequencing. The data analysis process begins with raw data, followed by quality control and optimization, OUT clustering, and then data analysis and taxonomic annotation. This includes species classification and abundance analysis, sample diversity analysis, PICRUSt2 functional prediction analysis and species difference analysis. All data analysis is conducted on the Majorbio Cloud Platform (https://cloud.majorbio.com).

### 2.10 Network pharmacology analysis

Identify the pharmacologically active constituents of four compounds in GQD within the Traditional Chinese Medicine Systems Pharmacology (TCMSP) database (https://old.tcmsp-e.com/tcmsp.phpb), applying the selection thresholds of oral bioavailability (OB) ≥30% and drug-likeness (DL) ≥0.18. The targets of relevant active ingredients were obtained from the SwissTargetPrediction database (https://swisstargetprediction.ch/) and the Similarity ensemble approach (SEA) database (https://sea.bkslab.org/) (37). Utilized “Ulcerative colitis” as the keyword; relevant targets from GeneCards (https://www.genecards.org/) were collected. The intersection targets obtained from the Venn diagram were used to construct protein-protein interaction (PPI) networks for these targets via the STRING database (https://string-db.org/). Processed the protein-protein interaction (PPI) network using Cytoscape 3.10.3 software to identify and visualize intersecting targets. Subsequently, screened the top 20 core targets using the Maximum Clique Centrality (MCC) topological parameter from the cytoHubba plugin. Applied the David database (https://davidbioinformatics.nih.gov/) to identify Gene Ontology (GO) terms and Kyoto Encyclopedia of Genes and Genomes (KEGG) pathways through analysis of shared genes.

### 2.11 Immunohistochemical staining

Paraffin-embedded colon tissue sections were first dewaxed and rehydrated, followed by treatment with xylene and graded ethanol solutions. Antigen retrieval was performed using a sodium citrate solution via microwave processing. Endogenous peroxidase was blocked with 3% hydrogen peroxide for 20 min, after which sections were washed with PBS and blocked with 10% BSA at 4°C for 1 h. After aspirating the blocking solution, primary antibody incubation was performed overnight at 4°C. The following day, after rewarming and PBS washing, HRP-conjugated secondary antibody incubation was conducted at 37°C for 1 h. Develop with DAB, stop with PBS, and counterstain nuclei with haematoxylin. Following dehydration and clearing, mount sections in neutral resin for observation under light microscopy. Analyze positive areas using ImageJ-win64 software. For each section, randomly image five non-overlapping regions within a 100 × field of view; the mean optical density value serves as the target protein’s positive expression level for that section, (n=5).

### 2.12 Immunofluorescence

Paraffin-embedded colon sections were deparaffinized and rehydrated using an eco-friendly deparaffinizing solution (three cycles of 10 min each), followed by three cycles of 5-minute washes with absolute ethanol. Sections were then washed three times with PBS (5 min per wash). Antigen retrieval was performed using EDTA solution (microwave: medium heat for 8 min, stand for 8 min, low heat for 8 min), followed by three PBS washes (5 min each). Subsequently, block the sections by incubating overnight at 4°C in a humidified chamber. Incubate with primary and secondary antibodies (interrupted by PBS washes; secondary antibody incubation at 37°C for 1 h). Counterstain with DAPI for 5 min at room temperature in the dark. Mount sections using an anti-fluorescence quenching mounting medium before acquiring images, (n=3).

### 2.13 Statistical analysis

GraphPad Prism 9.0 software was used for data analysis and graphing. The statistical data are presented as mean ± SD. One-way analysis of variance (ANOVA) was used for comparisons among multiple groups, and the T-test was used for comparisons between two groups. A *P*-value of <0.05 was considered statistically significant. *P < 0.05; **P < 0.01.

## 3 Results

### 3.1 Quality control analysis of GQD

**Figure 2:**
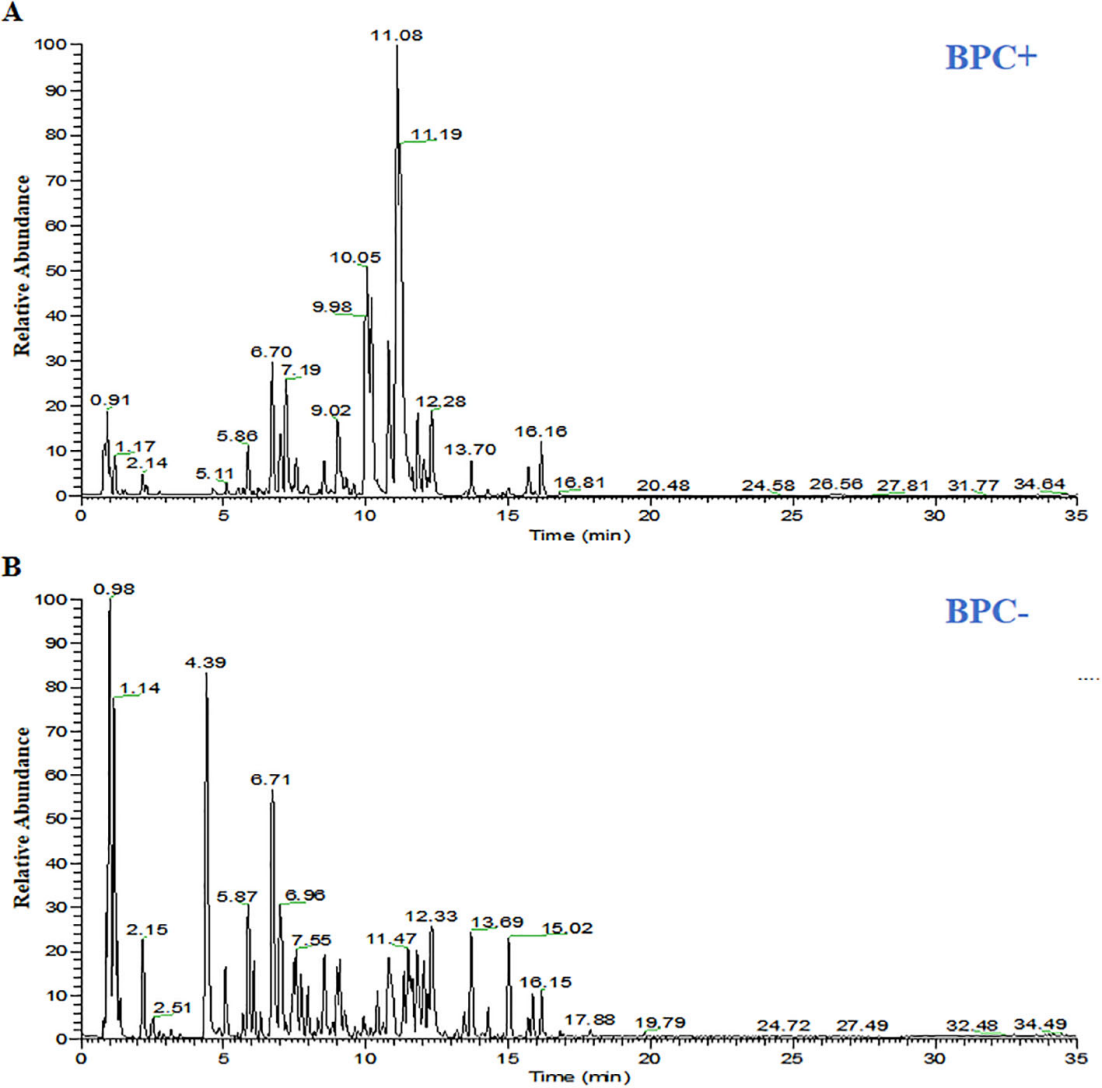
Base peak chromatogram of GQD by UPLC-QE-Orbitrap-MS. **(A)** The mode of positive; **(B)** The mode of negative.

Chemical profiling of GQD identified 71 major constituents using UPLC-QE-Orbitrap-MS in both positive and negative ion modes (**Figures 2A, B**; **Supplementary Table 2**). GQD, prepared by decocting Gegen, Huanglian, Huangqin, and Zhigancao, was found to be rich in puerarin, 3’-methoxypuerarin, daidzin, baicalin, wogonoside, berberine, palmatine, coptisine, and glycyrrhizic acid. All eight representative marker compounds were detected, indicating that the prepared decoction met established quality control standards.

### 3.2 GQD Ameliorates symptoms of DSS-induced colitis in mice

**Figure 3:**
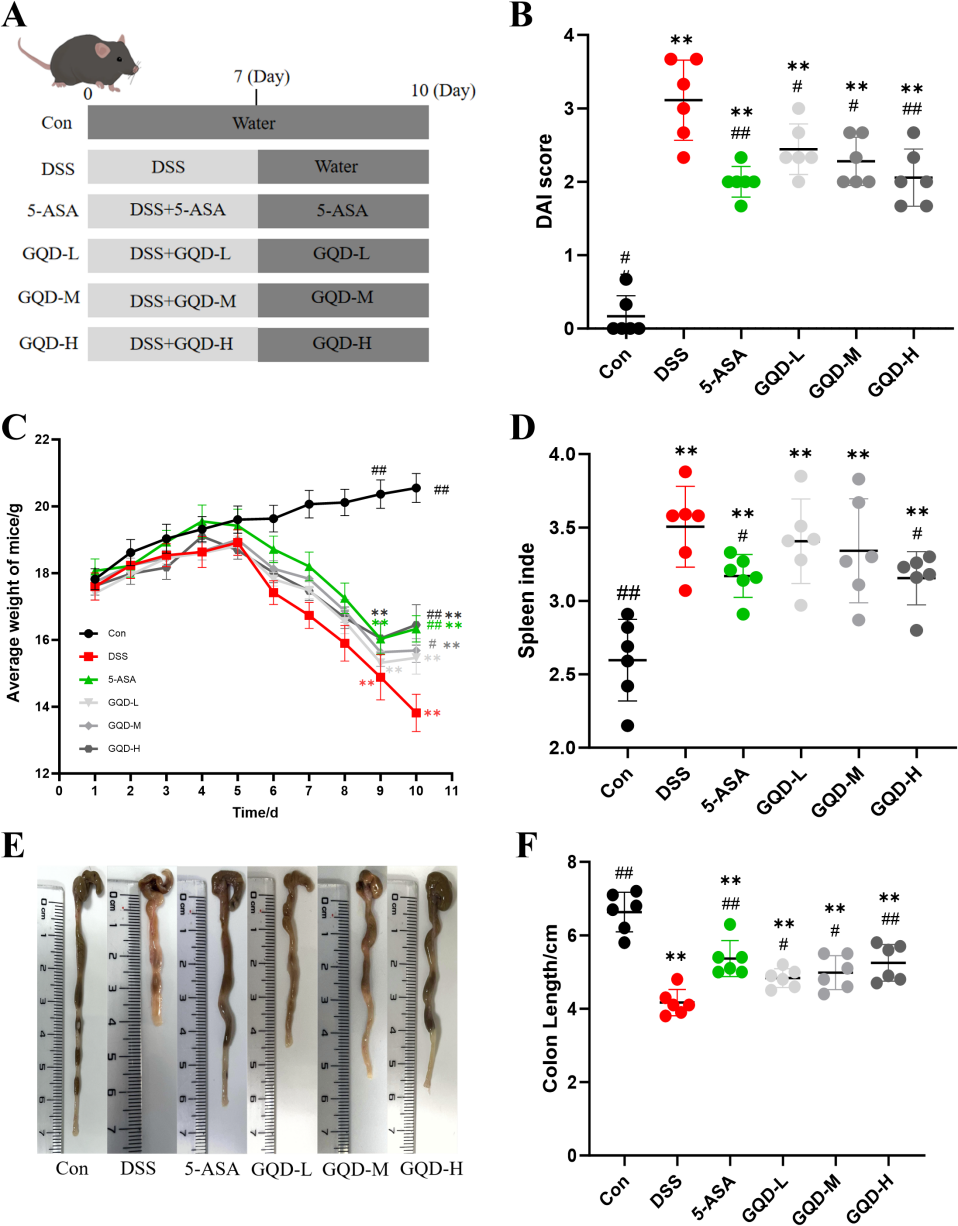
GQD ameliorates symptoms in UC mice. **(A)** Experimental Flowchart; **(B)** DAI score; **(C)** Body weight change; **(D)** Spleen index; **(E)** Colonic morphology; **(F)** colon length. Compared with the Con group: *P < 0.05, **P < 0.01. Compared with the DSS group: #*P* < 0.05, ##*P* < 0.01, ###*P* < 0.001, (n=6).

**Figure 3A** presents the flowchart of the DSS-induced UC mouse model. Throughout the experiment, DSS-treated mice exhibited pronounced body weight loss, loose stools, and overt rectal bleeding, leading to significantly elevated DAI scores compared with the Con group (**Figures 3B, C**). Both 5-ASA and GQD treatment significantly alleviated disease severity, as evidenced by reduced DAI scores and attenuated weight loss. Notably, the therapeutic efficacy observed in the GQD-H group was comparable to that in the 5-ASA group (**Figures 3B, C**).

In addition, the DSS group displayed a markedly increased spleen index and shortened colon length, consistent with systemic inflammation and colon injury. GQD treatment significantly normalized both spleen index and colon length in a dose-dependent manner (**Figures 3D–F**).

### 3.3 GQD reduces levels of inflammatory cytokines in the serum of UC mice

**Figure 4:**
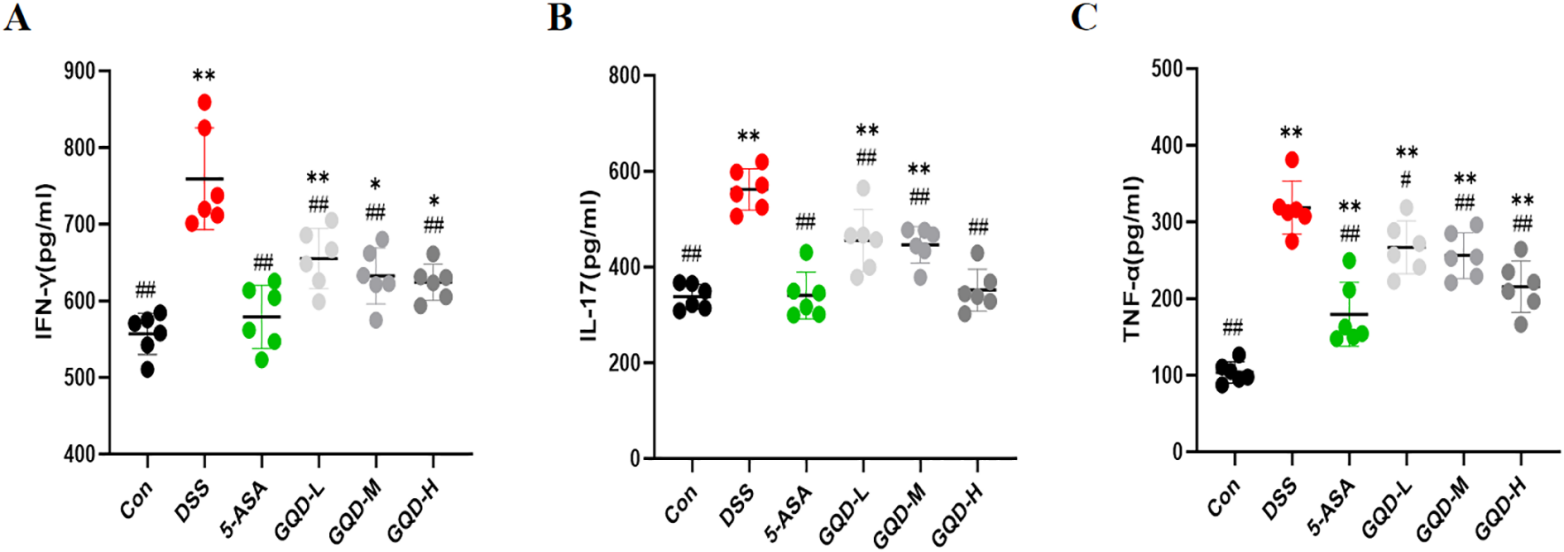
Levels of inflammatory cytokines in mouse serum. **(A)** IFN-γ; **(B)** IL-17; **(C)** TNF-α. Compared with the Con group: *p < 0.05, **p < 0.01. Compared with the DSS group: #*p* < 0.05, ##*p* < 0.01, (n=6).

To assess the systemic anti-inflammatory effect of GQD, we measured the serum levels of key pro-inflammatory cytokines. Compared with the Con group, mice in the DSS-induced UC model exhibited significantly elevated levels of IFN-γ, IL-17, and TNF-α (**Figures 4A–C**). This robust increase confirmed the successful induction of systemic inflammation. However, this elevation was markedly reversed by treatment with either the positive control drug 5-ASA or GQD, as evidenced by significantly reduced serum concentrations of these cytokines (**Figures 4A–C**). Importantly, the suppressive effect of GQD on these inflammatory mediators displayed a clear dose-dependent pattern, with higher doses eliciting more pronounced reductions.

### 3.4 GQD attenuates histopathological damage in the colonic tissue of UC mice

**Figure 5:**
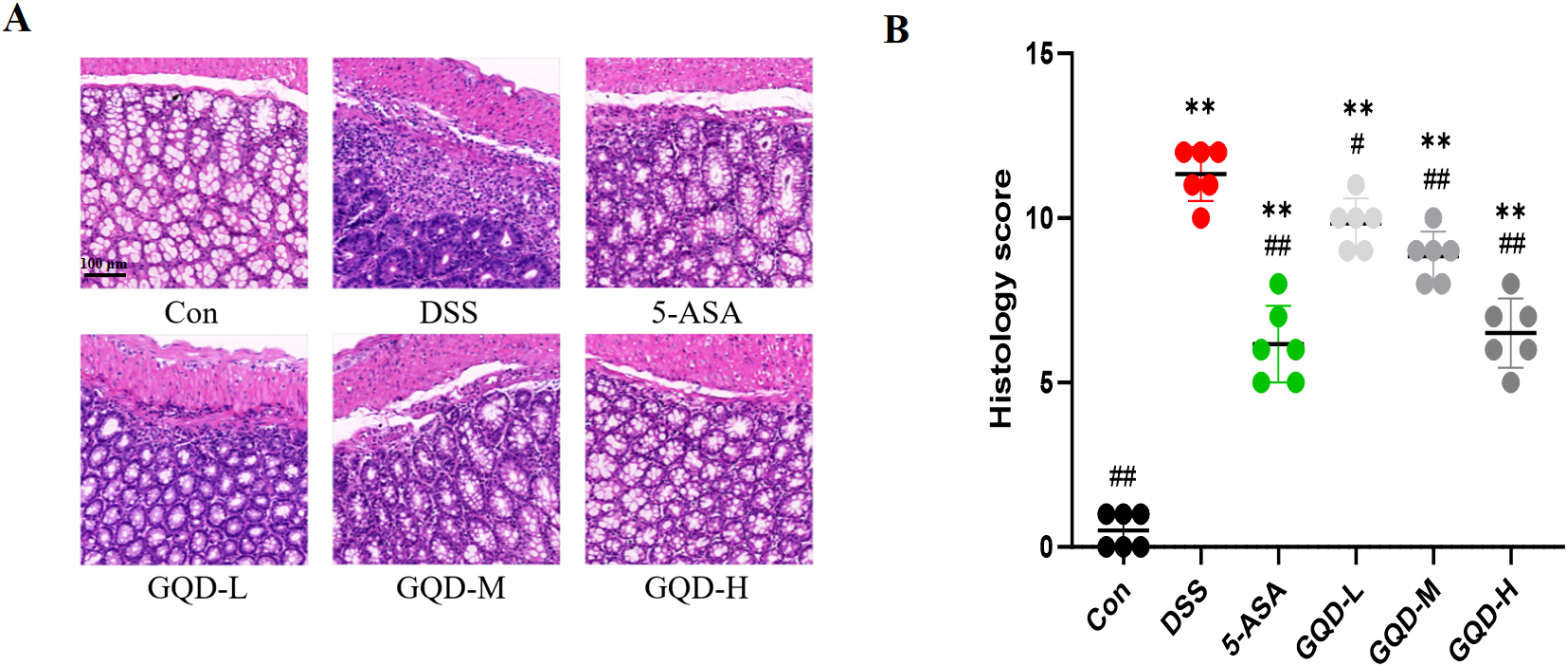
Histopathology of mouse colon tissue. **(A)** H&E staining, scale bar = 100 μm; **(B)** Histopathological score. Compared with the Con group: *p < 0.05, **p < 0.01. Compared with the DSS group: #*p* < 0.05, ##*p* < 0.01, (n=6).

The protective effect of GQD on colonic tissue was assessed by histopathological examination. Representative H&E-stained sections (**Figure 5A**) from the Con group displayed intact colonic structure, well-organized epithelial layers, absence of inflammatory infiltration, and preserved crypt architecture. In contrast, colons from DSS-treated mice exhibited severe ulceration characterized by the loss of the epithelial layer and exposure of the underlying lamina propria, extensive loss of goblet cells (evidenced by the near-complete absence of the characteristic clear, mucin-filled vacuoles), crypt destruction with distorted and shortened crypt structures, and marked inflammatory cell infiltration (dense infiltration of mononuclear and polymorphonuclear cells throughout the mucosa and submucosa), accompanied by substantial mucosal thickening.

Remarkably, treatment with GQD at various doses resulted in dose-dependent histological improvement, characterized by reduced inflammatory infiltration and partial restoration of mucosal architecture. A similar degree of improvement was observed in the 5-ASA positive control group. Consistently, quantitative histopathological scoring (**Figure 5B**) confirmed that GQD treatment significantly ameliorated DSS-induced tissue injury, with scores markedly lower than those in the DSS group. These findings collectively demonstrate that GQD exerts potent protective effects against DSS-induced colonic injury.

### 3.5 Non-targeted metabolomics reveals alterations in serum metabolite profiles of DSS-induced UC mice following GQD treatment

**Figure 6:**
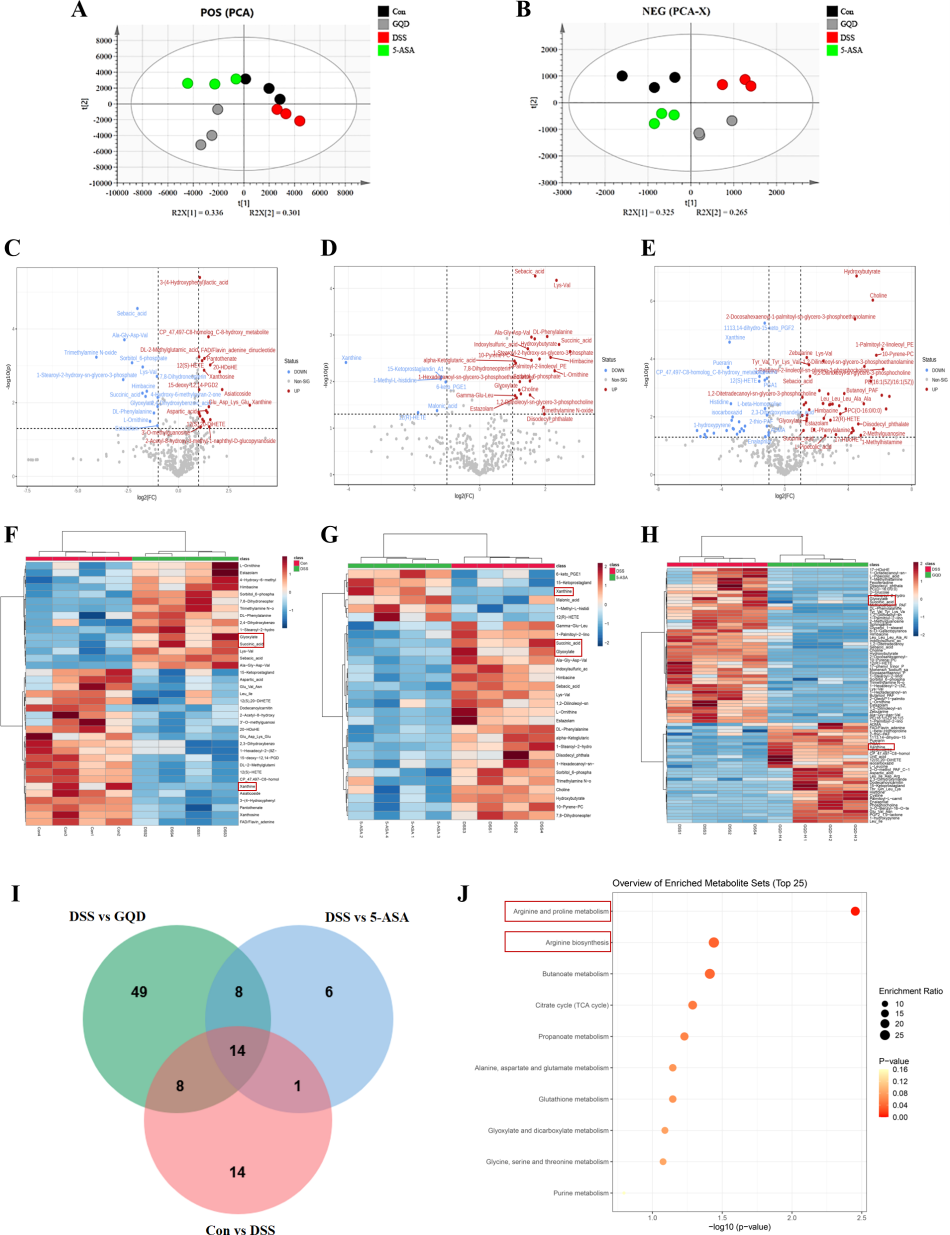
GQD treatment improves metabolic characteristics in UC mice. **(A)** Positive ion PCA plot; **(B)** Negative ion PCA plot; **(C-E)** Volcano plot of differential metabolites based on *FC* values and *P* values: Con vs DSS, DSS vs 5-ASA, DSS vs GQD, red dots indicate log_2_*FC* ≥ 1, blue dots indicate log_2_*FC* ≥ -1; **(F-H)** Differential metabolite Heatmap: Con vs DSS, DSS vs 5-ASA, DSS vs GQD; **(I)** Venn; **(J)** Bubble chart of KEGG enrichment analysis for differentially expressed metabolites.

Based on the aforementioned results, non-targeted metabolomics was employed to compare serum metabolic profiles among the Con, DSS, GQD-H, and 5-ASA groups. GQD−H was selected as the optimal efficacy group for metabolomic analysis. Principal component analysis (PCA) score plots demonstrated clear and reproducible separation among the four groups in both positive and negative ion modes (**Figures 6A, B**), indicating distinct metabolic states associated with disease induction and therapeutic intervention.

A total of 440 serum metabolites were identified. Compared with the DSS group, 37, 29, and 79 differentially expressed metabolites (DEMs) were identified in the Con, 5-ASA, and GQD groups, respectively (**Figures 6C–E**; **Supplementary Tables 3-S5**). Cluster heatmaps further confirmed that these DEMs robustly discriminated the compared groups (**Figures 6F–H**). Critically, cross-comparison revealed 14 DEMs shared across all three comparisons (Con vs DSS, 5-ASA vs DSS, and GQD vs DSS) (**Figure 6I**; **Supplementary Table 6**), including key metabolites such as glyoxylate, succinic acid, and xanthine. Notably, DSS-induced changes in these 14 metabolites were consistently reversed toward baseline levels following treatment with either GQD or 5-ASA, identifying them as core metabolites dysregulated in UC and responsive to therapeutic intervention.

Pathway enrichment analysis of these 14 key metabolites using MetaboAnalyst 5.0 revealed (**Figure 6J**; **Table 1**) that GQD primarily modulated arginine and proline metabolism and arginine biosynthesis. These are key sub-pathways of amino acid metabolism, critical for regulating anti-inflammatory responses in ulcerative colitis and closely linked to its pathogenesis. The purine metabolism pathway is closely associated with the major common differential metabolites succinic acid and xanthine. These findings indicate that GQD alleviates ulcerative colitis by repairing key metabolic dysregulation.

**Table 1:** Summary of related metabolic pathways.

No	Metabolite set	Total	Hits	Expect	P value
1	Arginine and proline metabolism	38	2	0.0997	0.00352
2	Arginine biosynthesis	14	1	0.0367	0.0363
3	Butanoate metabolism	15	1	0.0393	0.0388
4	Citrate cycle (TCA cycle)	20	1	0.0525	0.0515
5	Propanoate metabolism	23	1	0.0603	0.059
6	Alanine, aspartate and glutamate metabolism	28	1	0.0734	0.0715
7	Glutathione metabolism	28	1	0.0734	0.0715
8	Glyoxylate and dicarboxylate metabolism	32	1	0.0839	0.0814
9	Glycine, serine and threonine metabolism	33	1	0.0866	0.0839
10	Purine metabolism	65	1	0.17	0.16

### 3.6 GQD enhances gut microbiota diversity and alters microbial composition in UC mice

**Figure 7:**
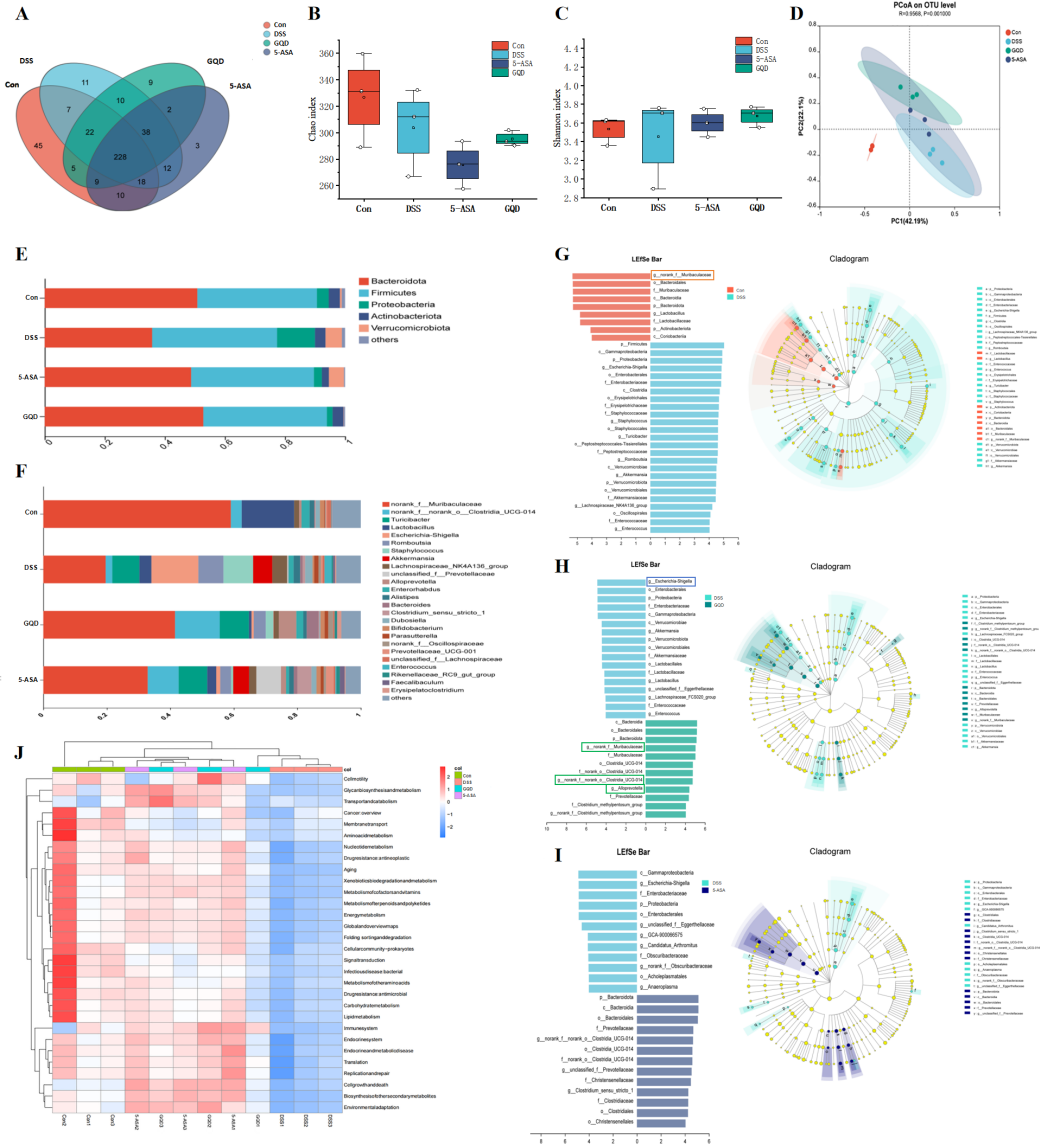
Gut microbiota diversity and composition. **(A)** Venn diagram of OTU distribution by gut florar; **(B, C)** Alpha diversity analysis: Chao, Shannon; **(D)** PCoA analysis; **(E)** Relative abundance of taxa at the phylum level; **(F)** Relative abundance of taxa at the Genus level; **(G-I)** Lefse analysis: Con vs DSS, DSS vs GQD, DSS vs 5-ASA (LDA≥4); **(J)** PICRUSt2 function prediction heat map.

To investigate the role of the gut microbiota in the therapeutic effect of GQD, we performed 16S rRNA sequencing on fecal samples. GQD−H was selected as the optimal efficacy group for 16S rRNA gene sequencing. Venn diagram analysis of OTUs identified a total of 429 OTUs, with 228 shared among all four groups (Con vs DSS = 275; Con vs 5-ASA = 296; DSS vs GQD = 298; all groups = 228; **Figure 7A**), indicating a core microbial community.

Beta diversity analysis showed a separation trend between the control group and other groups (**Figure 7D**). Notably, although the overall diversity indices showed no significant differences (**Figures 7B–D**), obvious changes were observed in specific bacterial taxa. At the phylum level (**Figure 7E**), Bacteroidetes, Firmicutes, and Proteobacteria remained dominant. Compared with the control group, the DSS group showed a significant increase in Proteobacteria and a marked decrease in Bacteroidetes. Compared with the DSS group, the GQD and 5−ASA groups exhibited significantly elevated Bacteroidetes and reduced Proteobacteria (**Supplementary Figure 1**), indicating that GQD effectively alleviated DSS−induced gut dysbiosis. At the genus level (**Figure 7F**), compared with the DSS group, the 5-ASA and GQD groups showed increased abundance of Bacteroides, norank_f:Muribaculaceae, norank_f:norank_o:Clostridia_UCG−014, Alloprevotella, and Bifidobacterium, as well as decreased abundance of Staphylococcus, Akkermansia, Romboutsia, and Escherichia−Shigella. Notably, GQD−H treatment significantly reduced potential pathogenic genera such as Escherichia−Shigella and increased beneficial genera including norank_f:Muribaculaceae (**Supplementary Figure 1**). These results demonstrated that both 5−ASA and GQD ameliorated gut dysbiosis at the phylum and genus levels.

Through LEfSe analysis (LDA>4), norank_f:Muribaculaceae and Lactobacillus were identified as the signature microbiota of the Con group, whilst the model group exhibited enrichment of Escherichia-Shigella pathogens. Notably, high-dose GQD treatment was characterized by norank_f:Muribaculaceae, norank_f:norank_o:Clostridia_UCG-014, Alloprevotella, and norank_f:Clostridium_methylpentosum_group (**Figures 7G–I**).

To further predict potential metabolic functions associated with the gut microbiota and to preliminarily explore the underlying mechanisms linking gut dysbiosis to DSS-induced UC, PICRUSt2 functional prediction analysis was performed on 16S rRNA sequencing data (**Figure 7J**; **Supplementary Figure 2**). The results showed that the DSS group primarily downregulated metabolic pathways such as amino acid biosynthesis, alanine, aspartate, and glutamate metabolism, purine metabolism, and pyrimidine metabolism. GQD and 5-ASA interventions significantly upregulated the abundances of these metabolic pathways. This indicates that the amelioration of UC by GQD is associated with the upregulation of key microbial metabolic functions, which may contribute to the restoration of host metabolic homeostasis.

### 3.7 Integrated multi-omics analysis explores the mechanism of action of GQD in treating UC

**Figure 8:**
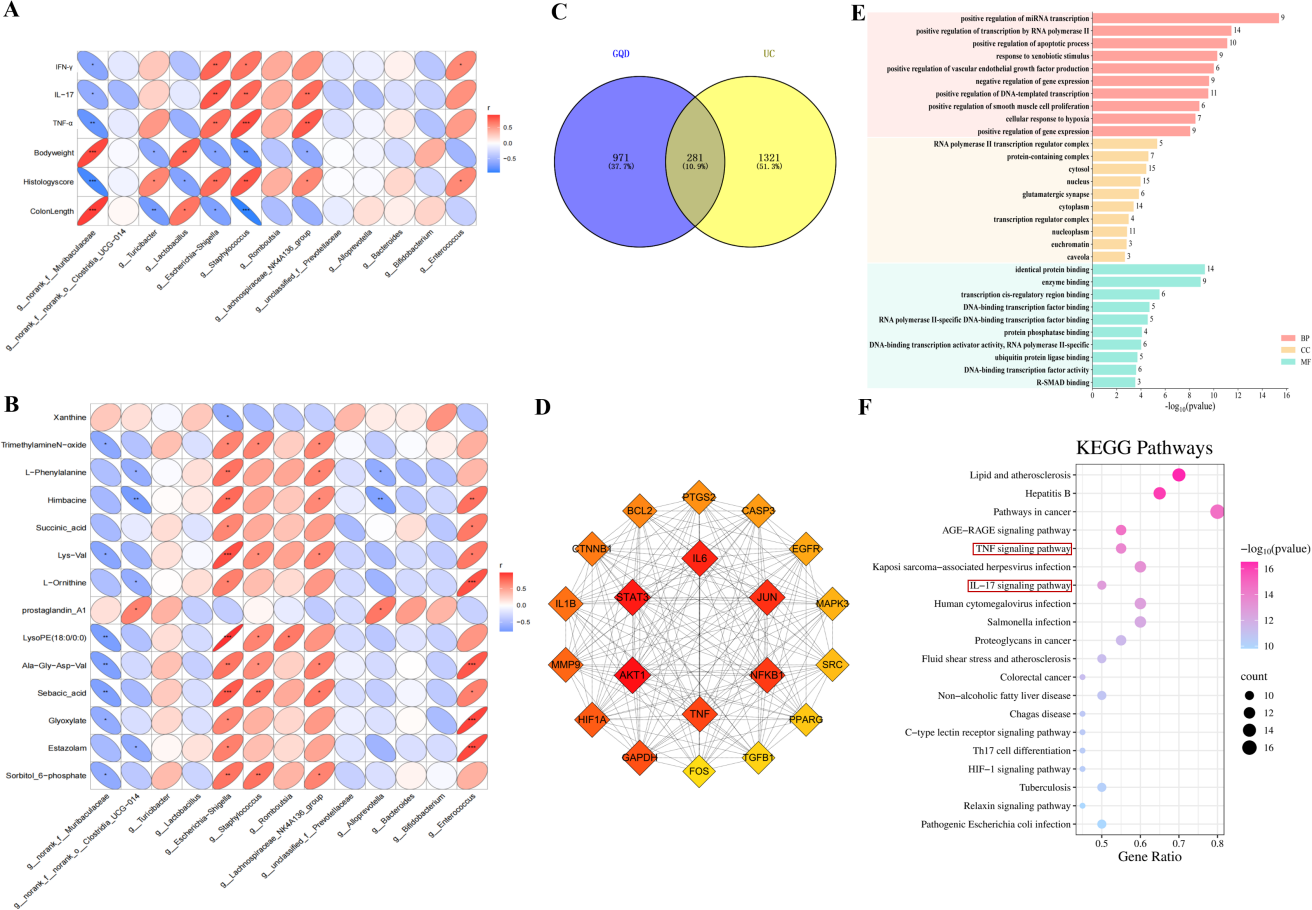
Correlation analysis combined with network pharmacology explores the mechanism of action of GQD in treating UC. **(A)** Correlation analysis between efficacy indicators and differential flora; **(B)** Correlation analysis between shared differential metabolites and differential flora; **(C)** Venn diagram. **(D)** MCC screening of the top 20 key genes’ PPI subnetwork. **(E)** GO enrichment analysis. **(F)** KEGG pathway enrichment analysis; *P < 0.05, **P < 0.01; ***P < 0.001.

To synthesize our findings and explore the potential mechanism of GQD, this study used correlation analysis and network pharmacology to examine the relationships among gut microbiota, serum metabolites, and inflammatory responses. Analysis of therapeutic indicators and gut microbiota (**Figure 8A**) shows beneficial bacteria norank_f:Muribaculaceae negatively correlate with serum inflammatory cytokines (IL-17, TNF-α, IFN-γ) and histopathological scores but positively with body weight and colon length. Conversely, pathogenic bacteria Escherichia-Shigella and Staphylococcus exhibit opposite correlations. A negative trend was observed between Lactobacillus abundance and pro−inflammatory cytokines (IFN−γ, IL−17, TNF−α). Although not statistically significant, metabolomic data suggest that Lactobacillus may function via regulating metabolic pathways rather than taxonomic abundance alone (**Figure 6J**; **Supplementary Figure 3**). The correlation analysis results between serum differential metabolites and differential microbial communities showed (**Figure 8B**) that g:norank_f:Muribaculaceae, Glyoxylate, and Sebacic acid negatively correlated; Escherichia-Shigella positively correlated with Glyoxylate, Sebacic acid, and Succinic acid, and negatively with Xanthine; Staphylococcus positively correlated with Sebacic acid. Using database searches, 1252 GQD bioactive compound targets and 1602 UC-related targets (median score ≥3.76) were identified. A Venn diagram (**Figure 8C**) revealed 281 shared targets, forming a PPI network with 280 nodes and 2,518 edges (**Supplementary Figure 4**). MCC screening highlighted 20 key targets (**Figure 8D**), including IL-6, TNF, AKT1, and STAT3, involved in inflammation. These top 20 core targets underwent KEGG and GO enrichment analysis via the DAVID database (https://davidbioinformatics.nih.gov/). GO analysis identified 280 biological processes (BP), 39 molecular functions (MF), and 31 cellular components (CC) terms (**Figure 8E**). KEGG enrichment analysis identified 132 signalling pathways, with IL-17 and TNF signalling pathways being particularly prominent (**Figure 8F**). These pathways are intrinsically linked to inflammatory responses, thereby providing a direction for subsequent mechanistic studies investigating GQD’s role in UC.

Integrated multi-omics analysis indicates that GQD exerts a pivotal role via the gut microbiota, regulating serum amino acid and purine metabolism to alleviate symptoms of UC in mice. This promotes metabolic normalization in the host and critically reduces systemic and local tissue inflammation.

### 3.8 GQD inhibits the expression of TNF-α, IFN-γ and IL-17 proteins in the colon of UC mice

**Figure 9:**
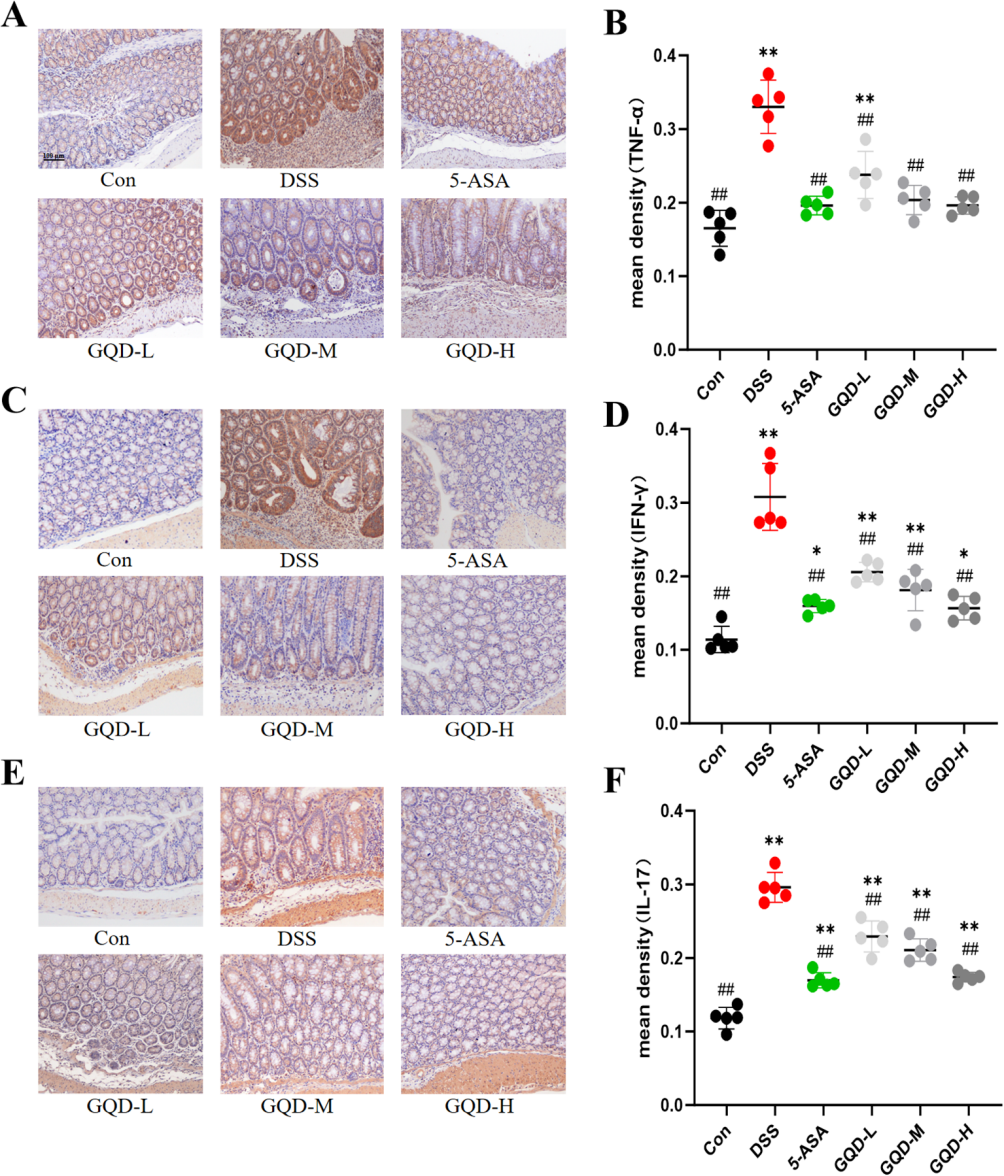
Expression of inflammatory cytokines in mouse colon. Scale bar = 100 μm. **(A)** TNF-α immunohistochemical staining; **(B)** TNF-α protein expression levels; **(C)** IFN-γ immunohistochemical staining; **(D)** IFN-γ protein expression levels; **(E)** IL-17 immunohistochemical staining; **(F)** IL-17 protein expression. Compared with the Con group: *p < 0.05, **p < 0.01; Compared with the DSS group: #*p* < 0.05, ##*p* < 0.01, (n = 6).

To experimentally validate the inflammatory regulatory mechanism of GQD, immunohistochemistry (IHC) was used to assess whether GQD suppresses the colonic expression of key pro-inflammatory cytokines. As shown in **Figures 9A–F**, DSS-induced UC resulted in severe epithelial damage, crypt loss, ulceration, and substantial inflammatory infiltration, accompanied by markedly elevated expression of TNF-α, IFN-γ, and IL-17 in colonic tissues.

Following GQD treatment, significant improvements were observed, including reduced epithelial damage, partial crypt restoration, and decreased inflammatory cell accumulation. Notably, GQD administration dose-dependently diminished the protein levels of TNF-α, IFN-γ, and IL-17, similar to the effects of 5-ASA. These results demonstrate that GQD effectively suppresses colonic inflammation by downregulating key pro-inflammatory mediators.

### 3.9 GQD regulates γδT cell activity in UC mice intestines, suppressing pro-inflammatory cytokines IL-17 and IFN-γ secretion

**Figure 10:**
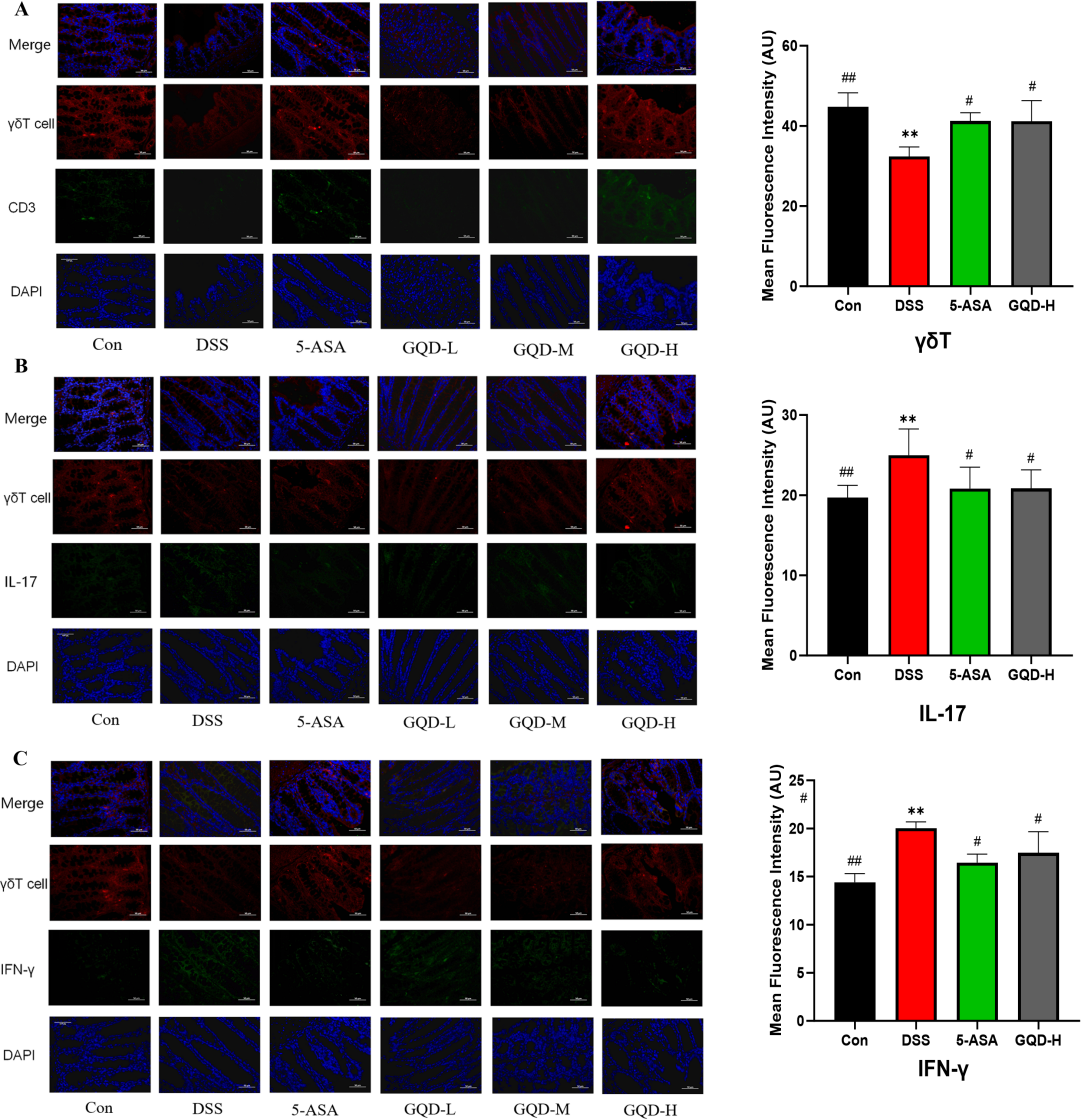
Immunofluorescence analysis of IFN-γ and IL-17 secretion by γδT cells in the colon. **(A)** Representative images of γδT (left) and quantitative analysis of their mean fluorescence intensity (right). **(B)** Representative images of IL-17+γδT (left) and quantitative analysis of their mean fluorescence intensity (right). **(C)** Representative images of IFN-γ+γδT (left) and quantitative analysis of their mean fluorescence intensity (right); Scale bar = 50 μm, Compared with the Con group: *p < 0.05, **p < 0.01; Compared with the DSS group: #p < 0.05, ##p < 0.01, (n = 3).

To further elucidate the cellular immune mechanism underlying the anti-inflammatory effects of GQD, immunofluorescence was performed to assess the activity of colonic γδT cells, including IFN-γ- and IL-17-producing subsets (**Figures 10A–C**). Compared with the Con group, DSS-treated mice showed reduced γδT cell mean fluorescence intensity, accompanied by increased IFN-γ^+^γδT and IL-17^+^γδT mean fluorescence intensity, indicating γδT-cell-mediated pro-inflammatory activation in UC.

In contrast, both GQD and 5-ASA treatment enhanced the mean fluorescence intensity of total γδT cells while reducing that of IFN-γ^+^γδT and IL-17^+^γδT cell frequencies, demonstrating suppressed γδT-cell-derived proinflammatory responses. These findings suggest that GQD alleviates UC symptoms by restoring γδT cell homeostasis and restraining their pathogenic cytokine secretion.

## 4 Disscussion

Ulcerative colitis (UC) is a chronic bowel disease characterized by inflammation that involves the gut microbiota, host immunity, and external environmental factors (38). Its characteristic remitting-relapsing course poses substantial clinical challenges to disease control and sustained remission (39). Therefore, therapeutic strategies targeting both intestinal microbial dysbiosis and abnormal immune activation are urgently needed. Gegen Qinlian Decoction (GQD), a classic Chinese herbal medicine that has been used for thousands of years to treat intestinal disorders (38), exhibits promising therapeutic potential. In this study, we demonstrate that GQD markedly alleviates colitis symptoms by reshaping gut microbiota composition, modulating γδT-cell activity, and suppressing key inflammatory pathways, thereby improving disease outcomes in DSS-induced UC.

Dysregulated inflammatory cytokines are a key factor in the pathogenesis of UC (40). Elevated pro-inflammatory cytokines in UC trigger tissue damage, ulceration, and colonic inflammation (41). TNF-α, IL-17, and IFN-γ are important pro-inflammatory cytokines (42). TNF-α degrades and damages the host mucosa, promotes inflammatory cell infiltration and tissue destruction, and exacerbates the inflammatory response (43). IL-17 induces other pro-inflammatory cytokines (such as TNF-α and IL-6) and chemokines to amplify the inflammatory cascade (44). IFN-γ inhibits the proliferation of intestinal epithelial cells while promoting their apoptosis, increasing intestinal barrier permeability and disrupting its integrity (45); it also activates macrophages to secrete more pro-inflammatory cytokines, including TNF-α and IL-17. All these processes are associated with the onset and deterioration of colitis symptoms. In this study, the detection of serum from DSS-induced UC mice revealed a significant increase in the levels of IL-17, TNF-α, and IFN-γ. GQD treatment reversed this elevation trend, alleviated ulcers, inflammation, and hematochezia, thereby exerting a therapeutic effect on UC.

Serum metabolites, acting as signalling molecules, play a pivotal role in regulating inflammation within the pathogenesis of ulcerative colitis (46). In UC, succinic acid accumulation triggers excessive inflammation and impairs intestinal metabolic homeostasis (47). As a key intermediate in the tricarboxylic acid cycle, succinic acid regulates the precursor supply for arginine and proline metabolism (48). It has been confirmed that regulating arginine metabolism and synthesis pathways can exert anti-UC effects, improve amino acid metabolic homeostasis, and thereby alleviate intestinal inflammation (49). Whereas in the present study, GQD downregulated the levels of the major common differential metabolite succinate and improved its metabolic disorders, which is consistent with previous reports. Aberrant glyoxylate metabolism is closely associated with disturbed intestinal homeostasis (50). Besides succinic acid, we identified two additional differential metabolites: glyoxylate and xanthine. Compared with the DSS group, GQD treatment downregulated glyoxylate and upregulated xanthine. Glyoxylate regulates glycine, serine, and threonine metabolism, another key pathway in amino acid metabolism. Glycine is synthesized from serine and threonine and is a non-essential amino acid. It negatively regulates TNF-α production and positively promotes IL-10 production. In mouse models of UC, the levels of glycine and threonine are significantly decreased, indicating disturbed glycine, serine, and threonine metabolism (51). Xanthine is the direct precursor of uric acid. Through purine metabolism, xanthine is oxidized to uric acid under the action of xanthine oxidase. The occurrence of UC is often accompanied by increased uric acid levels. In the absence of xanthine oxidase, xanthine cannot be converted to uric acid, leading to decreased xanthine content (52). GQD’s ability to upregulate xanthine suggests that it may regulate purine metabolism, a pathway closely associated with UC progression. In the present study, these three metabolites (succinate, glyoxylate, and xanthine) were found to intersect with multiple pathways (e.g., tricarboxylic acid cycle, amino acid biosynthesis, and purine metabolism), highlighting their central role in the therapeutic effect of GQD. GQD intervention significantly reversed the changes in these metabolites, restoring the levels of succinate, glyoxylate, xanthine, and other metabolites to be close to those in the Con group mice.

The gut microbiota plays a crucial role in regulating inflammatory responses in the human body and serves as a key player in the pathogenesis of UC (53). Firmicutes and Bacteroidetes are the dominant microbial phyla in the human gut. Studies have shown that an increased Firmicutes/Bacteroidetes (F/B) ratio is a marker of gut microbiota dysbiosis and a diagnostic indicator for UC (54). In the present study, GQD intervention restored the F/B ratio to the level of the Con group, which is consistent with previous reports. Overgrowth of Proteobacteria impairs the intestinal barrier and exacerbates inflammation (55), while the abundance of Verrucomicrobia is negatively correlated with disease activity (56). This study found that GQD reduced the abundance of Proteobacteria and improved gut microbiota composition. Although the observed increase in Verrucomicrobia abundance was contrary to some previous findings, GQD intervention normalized its level to that of the Con group mice. Alloprevotella produces propionate by decomposing dietary fiber, participates in regulating intestinal barrier function and immune homeostasis (57), and reduces pro-inflammatory factors such as TNF-α and IL-17, thereby creating conditions for intestinal barrier repair (58). Studies have shown that in UC models, the relative abundance of probiotics such as Alloprevotella is reduced, whereas the abundance of the pathogenic genus Escherichia-Shigella is elevated. This gut microbiota dysbiosis promotes UC development (59). Norank_f:Muribaculaceae, a core beneficial gut bacterium, produces anti-inflammatory metabolites such as short-chain fatty acids (SCFAs) by degrading dietary fiber and mucin (60). These SCFAs further enhance the mucosal barrier, inhibit macrophage activation, and reduce the secretion of TNF-α and IL-17 (61), which aligns with the research conclusion that increased abundance of norank_f:Muribaculaceae alleviates chronic inflammation. Additionally, norank_f:norank_o_Clostridia_UCG-014 positively regulates the growth of beneficial bacteria and negatively regulates the growth of pathogenic bacteria, which helps improve the diversity of gut microbiota (62). LEfSe analysis in this study revealed that the dominant bacteria in the Con group were norank_f:Muribaculaceae and Lactobacillus, while Escherichia-Shigella was the dominant genus in the DSS-induced UC group. After GQD intervention, the abundances of norank_f:Muribaculaceae, norank_f:norank_o_Clostridia_UCG-014, and Alloprevotella were restored. These microbial community changes are consistent with the LEfSe analysis results in this study.

Concurrently, the correlation analysis in this study further elucidates that GQD improves gut microbiota dysbiosis by ‘promoting the growth of beneficial bacteria and inhibiting the proliferation of pathogenic bacteria,’ regulates amino acid and purine metabolism, and suppresses serum inflammatory cytokines IFN-γ, IL-17, and TNF-α, thereby restoring intestinal microecological health. Previous mechanisms of GQD treatment for UC have primarily centred on the IL-17 signalling pathway, TNF signalling pathway, AGE-RAGE signalling pathway in diabetic complications, relaxin signalling pathway, fluid shear stress, and atherosclerosis (25), consistent with the enrichment results of this study. Given that the correlation analysis in this study strongly associates with gut microbiota and inflammatory cytokines such as TNF-α, IL-17, and IFN-γ, and network pharmacology reveals significant enrichment in the IL-17 signalling pathway and TNF signalling pathway, subsequent immunohistochemical detection of TNF, IL-17, and IFN-γ was performed in the colons of UC mice.

Gut microbiota dysbiosis disrupts the intestinal immune system, triggering excessive secretion of pro-inflammatory cytokines that cause intestinal damage (63). This damage, in turn, lowers intestinal γδT cell counts and impairs immune function (64). As both inflammatory and immunoregulatory factors, IFN-γ, IL-17, and TNF-α collectively maintain intestinal immune homeostasis. IFN-γ is mainly secreted by monocytes, Th1 cells, and γδT cells (65); IL-17 is predominantly produced by NK cells, Th17 cells, γδT cells, and CD8^+^ T cells (66); and TNF-α is primarily secreted by macrophages, NK cells, γδT cells, and CD4^+^ T cells (67). Notably, the secretion of these three cytokines is closely associated with γδT cells. Previous studies have shown that γδT cells play a crucial role in the body’s immune response and act as key drivers of inflammation (68). Specifically, γδT cells can secrete IL-2, IL-6, and IL-10 to activate B cells and suppress inflammatory responses (69). Conversely, IL-17^+^γδT cells and IFN-γ^+^γδ T cells secrete IL-17 and IFN-γ to potentiate inflammation, amplifying the inflammatory cascade (70). These findings indicate that γδT cells can modulate the immune microenvironment in mice and boost therapeutic efficacy against UC. Consistent with previous research, our results show that GQD intervention significantly reduced IFN-γ and IL-17 secretion in colonic tissues, increased γδT cell numbers in the colonic mucosa, and decreased the proportions of IFN-γ^+^γδT cells and IL-17^+^γδT cells. Collectively, these effects enhance immune function and suppress inflammation, thereby alleviating UC symptoms.

This study confirms that GQD, a classical Chinese herbal formula, exerts a significant therapeutic effect on DSS-induced UC. Its mechanism primarily involves multi-dimensional regulation of the gut microbiota-immuno-metabolic network: enriching beneficial bacteria and suppressing pathogens to ameliorate gut dysbiosis; modulating γδT lymphocyte function by elevating intestinal mucosal γδT cell counts and reducing IFN-γ^+^γδT/IL-17^+^γδT subsets, thereby decreasing pro-inflammatory cytokine secretion; correcting metabolic disturbances by restoring amino acid and purine metabolic balance, as well as lowering the pro-inflammatory metabolite succinate. These synergistic regulatory effects suppress TNF-α, IL-17, and IFN-γ expression, mitigate intestinal inflammation, ulceration, and tissue damage, and restore intestinal microecological and immune homeostasis, providing experimental evidence for GQD’s multi-target synergistic therapeutic effects against UC.

Despite the strengths of this study, certain limitations should be noted. First, although γδT-cell-associated immune alterations were observed, the precise regulatory mechanisms remain incompletely defined. Second, although the sample size is limited, the samples are representative and the overall evidence chain is solid, supporting the reliability of the conclusions. Further studies with larger sample sizes are warranted.Third, although our findings suggest that GQD may regulate γδT-cell activity, the specific bioactive components responsible for these effects remain to be identified, necessitating further mechanistic studies.

## 5 Conclusion

**Figure 11:**
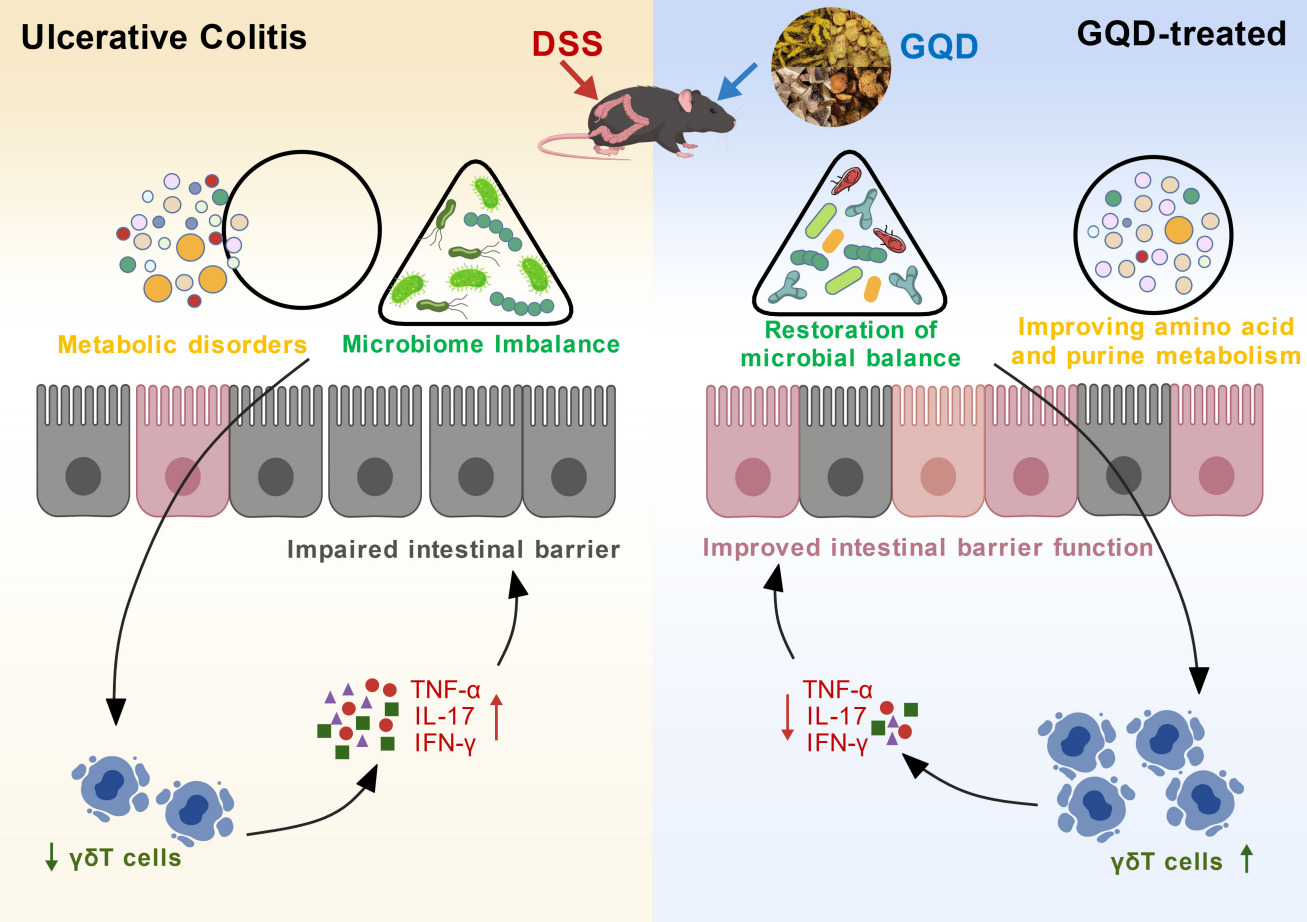
Mechanism diagram of GQD-mediated improvement in DSS-induced UC. (Created with BioGDP.com) (71).

In summary, GQD exerts therapeutic effects on UC by remodelling the composition and metabolic activity of the gut microbiota, thereby improving intestinal mucosal damage, modulating γδT cell-mediated immune responses, and influencing amino acid and purine metabolic pathways (**Figure 11**).

## Data Availability

The data presented in the study are deposited in the NCBI BioProject repository, accession number PRJNA1443306.

## References

[1] Neurath MF Leppkes M . Resolution of ulcerative colitis. Semin Immunopathol. (2019) 41:747–56. doi: 10.1007/s00281-019-00751-6 31278430

[2] Huang S Wang X Xie X Su Y Pan Z Li Y . Dahuang mudan decoction repairs intestinal barrier in chronic colitic mice by regulating the function of ILC3. J Ethnopharmacol. (2022) 299:115652. doi: 10.1016/j.jep.2022.115652 36038092

[3] Zheng Y Liang C Li Z Chen J Chen Z Jiang Y . Study on the mechanism of Huangqin decoction on rats with ulcerative colitis of damp-heat type base on mtDNA, TLR4, p-PI3K, p-Akt protein expression and microbiota. J Ethnopharmacol. (2022) 295:115356. doi: 10.1016/j.jep.2022.115356 35568112

[4] Zhou Y Wu J Wang H Feng W Peng F Zhang R . Fuzi lizhong pills alter microbial community compositions and metabolite profiles in ulcerative colitis rat with spleen-kidney yang deficiency syndrome. J Ethnopharmacol. (2024) 335:118645. doi: 10.1016/j.jep.2024.118645 39089661

[5] Qiao H He J Chen Y Jin F Huang Y Li Y . Sporoderm-broken of Ganoderma lucidum spore polysaccharides alleviate dextran sulfate sodium-induced colon inflammation in mice by regulating Th17/Treg homeostasis and restore gut microbiota balance. Int J Biol Macromol. (2025) 323:147015. doi: 10.1016/j.ijbiomac.2025.147015 40846013

[6] Richardson J Nelson L Curry A Ralston S . The increasing global incidence of ulcerative colitis; implications for the economic burden of ulcerative colitis. Value Health. (2018) 21:S82. doi: 10.1016/j.jval.2018.04.556 41842036

[7] Luo Y Zhu F Wu J Wu J Wu P Liu Y . Effect of Shenling Baizhu San on intestinal flora in a rat model of ulcerative colitis with spleen deficiency and dampness. Evid Based Complement Alternat Med. (2022) 2022:9985147. doi: 10.1155/2022/9985147 35190749 PMC8858063

[8] Merino JJ Rey NB Fernández-García R . Microbiota and gut inflammatory markers (Zonulin and fecal calprotectin) exhibit age-dependent variation in patients with ulcerative colitis. Life. (2025) 15:1412. doi: 10.3390/life15091412 41010354 PMC12471355

[9] Tian L Gao H Yao T Chen Y Gao L Han J . Interactions between NAD+ metabolism and immune cell infiltration in ulcerative colitis: subtype identification and development of novel diagnostic models. Front Immunol. (2025) 16:1479421. doi: 10.3389/fimmu.2025.1479421 39975557 PMC11835821

[10] Tang X Huang Y Zhu Y Xu Y . Immune dysregulation in ulcerative colitis: pathogenic mechanisms and therapeutic strategies of traditional Chinese medicine. Front Cell Dev Biol. (2025) 13:1610435. doi: 10.3389/fcell.2025.1610435 40538978 PMC12176777

[11] Caba L Florea A Cianga P Drug V Popescu R Mihai C . Genetic and epigenetic factors in ulcerative colitis: a narrative literature review. Genes. (2025) 16:1085. doi: 10.3390/genes16091085 41010030 PMC12470167

[12] Tse CS Yonan A Nguyen H Dulai P Singh S Dervieux T . Multiple environmental and social determinants of health factors were not associated with the gastrointestinal symptomatic burden of ulcerative colitis with mucosal healing. Inflammation Bowel Dis. (2023) 29:S44–5. doi: 10.1093/ibd/izac247.081 40487282

[13] Peery AF Khalili H Münch A Pardi DS . Update on the epidemiology and management of microscopic colitis. Clin Gastroenterol Hepatol. (2025) 23:490–500. doi: 10.1016/j.cgh.2024.08.026 39270919 PMC11825284

[14] Li S Zhang Y Li K Liu Y Chi S Wang Y . Update on the pathogenesis of the Hirschsprung-associated enterocolitis. Int J Mol Sci. (2023) 24:4602. doi: 10.3390/ijms24054602 36902033 PMC10003052

[15] Keshteli AH Madsen KL Dieleman LA . Diet in the pathogenesis and management of ulcerative colitis; a review of randomized controlled dietary interventions. Nutrients. (2019) 11:1498. doi: 10.3390/nu11071498 31262022 PMC6683258

[16] Wang X Liu X Gao Q Gu X Zhang G Sheng Z . Gegen Qinlian decoction treatment of asymptomatic hyperuricemia by targeting circadian immune function. Chin Med. (2023) 18:77. doi: 10.1186/s13020-023-00775-z 37370132 PMC10304353

[17] Ga Y Wei Y Zhao Q Fan Y Zhang Y Zhang Z . Puerariae radix protects against ulcerative colitis in mice by inhibiting NLRP3 inflammasome activation. Food Sci Hum Wellness. (2024) 13:2266–76. doi: 10.26599/FSHW.2022.9250189 41311685

[18] Zhang Z Cui Y Ouyang H Zhu W Feng Y Yao M . Radix pueraria lobata polysaccharide relieved DSS-induced ulcerative colitis through modulating PI3K signaling. J Funct Foods. (2023) 104:105514. doi: 10.1016/j.jff.2023.105514 41842036

[19] Tao Q Liang Q Fu Y Qian J Xu J Zhu Y . Puerarin ameliorates colitis by direct suppression of macrophage M1 polarization in DSS mice. Phytomedicine. (2024) 135:156048. doi: 10.1016/j.phymed.2024.156048 39326132

[20] Li C Deng L Pu M Ye X Lu Q . Coptisine alleviates colitis through modulating gut microbiota and inhibiting TXNIP/NLRP3 inflammasome. J Ethnopharmacol. (2024) 335:118680. doi: 10.1016/j.jep.2024.118680 39117021

[21] Peng X Wang K Wang Y Lu Y Lv F Cui Y . Exploration of the mechanism of the control of coccidiosis in chickens based on network pharmacology and molecular docking with the addition of modified Gegen Qinlian decoction. Front Vet Sci. (2022) 9:849518. doi: 10.3389/fvets.2022.849518 35372563 PMC8968990

[22] Cai J Zhong X Liang J Xu C Yu H Xian M . Structural characterization, anti-inflammatory and glycosidase inhibitory activities of two new polysaccharides from the root of Pueraria lobata. RSC Adv. (2021) 11:35994–6006. doi: 10.1039/d1ra07385k 35492792 PMC9043251

[23] Wang Y Zhang J Zhang B Lu M Ma J Liu Z . Modified Gegen Qinlian decoction ameliorated ulcerative colitis by attenuating inflammation and oxidative stress and enhancing intestinal barrier function *in vivo* and *in vitro*. J Ethnopharmacol. (2023) 313:116538. doi: 10.1016/j.jep.2023.116538 37086872

[24] Wang X Huang S Zhang M Su Y Pan Z Liang J . Gegen Qinlian decoction activates AhR/IL-22 to repair intestinal barrier by modulating gut microbiota-related tryptophan metabolism in ulcerative colitis mice. J Ethnopharmacol. (2023) 302:115919. doi: 10.1016/j.jep.2022.115919 36356716

[25] Wei M Li H Li Q Qiao Y Ma Q Xie R . Based on network pharmacology to explore the molecular targets and mechanisms of Gegen Qinlian decoction for the treatment of ulcerative colitis. BioMed Res Int. (2020) 2020:5217405. doi: 10.1155/2020/5217405 33299870 PMC7710413

[26] Zhao Y Luan H Jiang H Xu Y Wu X Zhang Y . Gegen Qinlian decoction relieved DSS-induced ulcerative colitis in mice by modulating Th17/Treg cell homeostasis via suppressing IL-6/JAK2/STAT3 signaling. Phytomedicine. (2021) 84:153519. doi: 10.1016/j.phymed.2021.153519 33640781

[27] Yun X Zhang Q Fang Y Lv C Chen Q Chu Y . Madecassic acid alleviates colitis-associated colorectal cancer by blocking the recruitment of myeloid-derived suppressor cells via the inhibition of IL-17 expression in γδT17 cells. Biochem Pharmacol. (2022) 202:115138. doi: 10.1016/j.bcp.2022.115138 35700756

[28] Kathania M Khare P Zeng M Cantarel B Zhang H Ueno H . Itch inhibits IL-17-mediated colon inflammation and tumorigenesis by ROR-γt ubiquitination. Nat Immunol. (2016) 17:997–1004. doi: 10.1038/ni.3488 27322655

[29] Jung K-H Shin D Kim S Min D Kim W Kim J . Intratracheal ovalbumin administration induces colitis through the IFN-γ pathway in mice. Front Immunol. (2019) 10:530. doi: 10.3389/fimmu.2019.00530 30949176 PMC6437076

[30] Yang T Ma X Wang R Liu H Wei S Jing M . Berberine inhibits IFN-γ signaling pathway in DSS-induced ulcerative colitis. Saudi Pharm J. (2022) 30:764–78. doi: 10.1016/j.jsps.2022.03.015 35812150 PMC9257906

[31] Liu T Han Z Jia S Ma K Li M Yi X . An acidic polysaccharide from lycium barbarum L: isolation, purification, structural characterization, and therapeutic effects on ulcerative colitis. Int J Biol Macromol. (2025) 319:145602. doi: 10.1016/j.ijbiomac.2025.145602 40581006

[32] Wang W Li H Yang D Cai W Che H Li H . Study on the ameliorative effect of marine fungus hansfordia sinuosae extracellular polysaccharide on DSS-induced ulcerative colitis and depression-like behavior. Int J Biol Macromol. (2025) 309:142852. doi: 10.1016/j.ijbiomac.2025.142852 40188913

[33] Tao C Luo F Wang Y Gao X Cao Y Wang K . Mannose-modified prunus persica kernel protein nanoparticles loading baicalin coated with lycium barbarum polysaccharide for ulcerative colitis treatment. Carbohydr Polym. (2026) 371:124489. doi: 10.1016/j.carbpol.2025.124489 41198305

[34] Shu J Qin D Tu W Zhao D Yang Q Shao S . Single-cell and spatially resolved transcriptomics elucidate the therapeutic mechanism of tripterygium wilfordii polyglycosidium in ulcerative colitis. Phytomedicine. (2026) 150:157569. doi: 10.1016/j.phymed.2025.157569 41351984

[35] Song Q You X . Protective bioactivity of Ziziphus jujuba dates polysaccharide on colitis via improving the intestinal barrier and modifying the gut microbiota. Carbohydr Polym Technol Appl. (2025) 11:100931. doi: 10.1016/j.carpta.2025.100931 41842036

[36] Xu D Liu D Jiang N Xie Y He D Cheng J . Narirutin mitigates dextrose sodium sulfate-induced colitis in mice by modulating intestinal flora. Phytomed Int J Phytother Phytopharm. (2024) 130:155730. doi: 10.1016/j.phymed.2024.155730 38759313

[37] Keiser MJ Roth BL Armbruster BN Ernsberger P Irwin JJ Shoichet BK . Relating protein pharmacology by ligand chemistry. Nat Biotechnol. (2007) 25:197–206. doi: 10.1038/nbt1284 17287757

[38] Hu Y Tang J Xie Y Xu W Zhu W Xia L . Gegen Qinlian decoction ameliorates TNBS-induced ulcerative colitis by regulating Th2/Th1 and Tregs/Th17 cells balance, inhibiting NLRP3 inflammasome activation and reshaping gut microbiota. J Ethnopharmacol. (2024) 328:117956. doi: 10.1016/j.jep.2024.117956 38428658

[39] Fanizzi F Allocca M Fiorino G Zilli A Furfaro F Parigi TL . Raising the bar in ulcerative colitis management. Ther Adv Gastroenterol. (2024) 17:17562848241273066. doi: 10.1177/17562848241273066 39600566 PMC11589388

[40] Neurath MF . Cytokines in inflammatory bowel disease. Nat Rev Immunol. (2014) 14:329–42. doi: 10.1038/nri3661 24751956

[41] Neurath MF . Strategies for targeting cytokines in inflammatory bowel disease. Nat Rev Immunol. (2024) 24:559–76. doi: 10.1038/s41577-024-01008-6 38486124

[42] Feagan BG Rutgeerts P Sands BE Hanauer S Colombel J-F Sandborn WJ . Vedolizumab as induction and maintenance therapy for ulcerative colitis. N Engl J Med. (2013) 369:699–710. doi: 10.1056/NEJMoa1215734 23964932

[43] Leppkes M Roulis M Neurath MF Kollias G Becker C . Pleiotropic functions of TNF-α in the regulation of the intestinal epithelial response to inflammation. Int Immunol. (2014) 26:509–15. doi: 10.1093/intimm/dxu051 24821262

[44] Katz Y Nadiv O Beer Y . Interleukin-17 enhances tumor necrosis factor alpha-induced synthesis of interleukins 1,6, and 8 in skin and synovial fibroblasts: a possible role as a “fine-tuning cytokine” in inflammation processes. Arthritis Rheum. (2001) 44:2176–84. doi: 10.1002/1529-0131(200109)44:9<2176::aid-art371>3.0.co;2-4 11592383

[45] Langer V Vivi E Regensburger D Winkler TH Waldner MJ Rath T . IFN-γ drives inflammatory bowel disease pathogenesis through VE-cadherin–directed vascular barrier disruption. J Clin Invest. (2019) 129:4691–707. doi: 10.1172/JCI124884 31566580 PMC6819119

[46] Li M Zhang R Xin M Xu Y Liu S Yu B . Discovery and validation of potential serum biomarkers with pro-inflammatory and DNA damage activities in ulcerative colitis: a comprehensive untargeted metabolomic study. Metabolites. (2022) 12:997. doi: 10.3390/metabo12100997 36295899 PMC9609580

[47] Chen L Xie L Wang L Zhan X Zhuo Z Jiang S . Patchoulene epoxide mitigates colitis and hepatic damage induced by dextran sulfate sodium by regulating the colonic microbiota and purine metabolism. Front Immunol. (2025) 16:1509114. doi: 10.3389/fimmu.2025.1509114 40028318 PMC11868103

[48] Dai M Bu S Miao Z . Succinate metabolism: underlying biological mechanisms and emerging therapeutic targets in inflammatory bowel disease. Front Immunol. (2025) 16:1630310. doi: 10.3389/fimmu.2025.1630310 41000375 PMC12457111

[49] Liu S Sun H Du Z Lu S Wang C Zhang Y . Metabolomics and proteomics reveal blocking argininosuccinate synthetase 1 alleviates colitis in mice. Nat Commun. (2025) 16:6983. doi: 10.1038/s41467-025-62217-8 40739098 PMC12311138

[50] McQueen P Busman-Sahay K Rieder F Noël-Romas L McCorrister S Westmacott G . Intestinal proteomic analysis of a novel non-human primate model of experimental colitis reveals signatures of mitochondrial and metabolic dysfunction. Mucosal Immunol. (2019) 12:1327–35. doi: 10.1038/s41385-019-0200-2 31481749 PMC7673647

[51] Xie D Li F Pang D Zhao S Zhang M Ren Z . Systematic metabolic profiling of mice with dextran sulfate sodium-induced colitis. J Inflammation Res. (2021) 14:2941–53. doi: 10.2147/JIR.S313374 34239317 PMC8259941

[52] Wigner P Grębowski R Bijak M Szemraj J Saluk-Bijak J . The molecular aspect of nephrolithiasis development. Cells. (2021) 10:1926. doi: 10.3390/cells10081926 34440695 PMC8393760

[53] Le Berre C Honap S Peyrin-Biroulet L . Ulcerative colitis. Lancet. (2023) 402:571–84. doi: 10.1016/S0140-6736(23)00966-2 37573077

[54] Zhu S Han M Liu S Fan L Shi H Li P . Composition and diverse differences of intestinal microbiota in ulcerative colitis patients. Front Cell Infect Microbiol. (2022) 12:953962. doi: 10.3389/fcimb.2022.953962 36111238 PMC9468541

[55] Khorsand B Asadzadeh Aghdaei H Nazemalhosseini-Mojarad E Nadalian B Nadalian B Houri H . Overrepresentation of Enterobacteriaceae and Escherichia coli is the major gut microbiome signature in Crohn’s disease and ulcerative colitis; a comprehensive metagenomic analysis of IBDMDB datasets. Front Cell Infect Microbiol. (2022) 12:1015890. doi: 10.3389/fcimb.2022.1015890 36268225 PMC9577114

[56] Michail S Durbin M Turner D Griffiths AM Mack DR Hyams J . Alterations in the gut microbiome of children with severe ulcerative colitis. Inflammation Bowel Dis. (2012) 18:1799–808. doi: 10.1002/ibd.22860 22170749 PMC3319508

[57] Han B Shi L Bao M-Y Yu F-L Zhang Y Lu X-Y . Dietary ellagic acid therapy for CNS autoimmunity: Targeting on Alloprevotella rava and propionate metabolism. Microbiome. (2024) 12:114. doi: 10.1186/s40168-024-01819-8 38915127 PMC11194905

[58] Zhang S Zhou R Xie X Xiong S Li L Li Y . Polysaccharides from Lycium barbarum, yam, and sunflower ameliorate colitis in a structure and intrinsic flora-dependent manner. Carbohydr Polym. (2025) 349:122905. doi: 10.1016/j.carbpol.2024.122905 39643421

[59] Xie Q Li H Ma R Ren M Li Y Li J . Effect of Coptis chinensis franch and Magnolia officinalis on intestinal flora and intestinal barrier in a TNBS-induced ulcerative colitis rats model. Phytomed Int J Phytother Phytopharm. (2022) 97:153927. doi: 10.1016/j.phymed.2022.153927 35030387

[60] Chamaillard M Cesaro A Lober P-E Hober D . Decoding norovirus infection in Crohn’s disease. Inflammation Bowel Dis. (2014) 20:767–70. doi: 10.1097/01.MIB.0000440613.83703.4a 24351661

[61] Vinolo MAR Rodrigues HG Nachbar RT Curi R . Regulation of inflammation by short chain fatty acids. Nutrients. (2011) 3:858–76. doi: 10.3390/nu3100858 22254083 PMC3257741

[62] Li X Qiao G Chu L Lin L Zheng G . Smilax China L. polysaccharide alleviates dextran sulphate sodium-induced colitis and modulates the gut microbiota in mice. Foods. (2023) 12:1632. doi: 10.3390/foods12081632 37107427 PMC10137970

[63] Giuffrida P Corazza GR Di Sabatino A . Old and new lymphocyte players in inflammatory bowel disease. Dig Dis Sci. (2018) 63:277–88. doi: 10.1007/s10620-017-4892-4 29275447

[64] Dart RJ Zlatareva I Vantourout P Theodoridis E Amar A Kannambath S . Conserved γδ T cell selection by BTNL proteins limits progression of human inflammatory bowel disease. Science. (2023) 381:eadh0301. doi: 10.1126/science.adh0301 37708268 PMC7615126

[65] Jiang Y Ye L Sheng C Zhu J Xu J Cheng X . Similarities and differences in the immune characteristics of intestinal gamma delta T cells from patients with Crohn’s disease and ulcerative colitis and their correlation with disease activity. Immun Inflammation Dis. (2025) 13:e70273. doi: 10.1002/iid3.70273 41090206 PMC12521879

[66] Ma H Yuan Y Zhao L Ye Z Xu J Li M . Association of γδ T cell compartment size to disease activity and response to therapy in SLE. PloS One. (2016) 11:e0157772. doi: 10.1371/journal.pone.0157772 27333282 PMC4917177

[67] Gordon JR Galli SJ . Mast cells as a source of both preformed and immunologically inducible TNF-alpha/cachectin. Nature. (1990) 346:274–6. doi: 10.1038/346274a0 2374592

[68] Bank I Duvdevani M Livneh A . Expansion of gammadelta T-cells in Behçet’s disease: role of disease activity and microbial flora in oral ulcers. J Lab Clin Med. (2003) 141:33–40. doi: 10.1067/mlc.2003.1 12518166

[69] Liu N Qin H Cai Y Li X Wang L Xu Q . Dynamic trafficking patterns of IL-17-producing γδ T cells are linked to the recurrence of skin inflammation in psoriasis-like dermatitis. EBioMedicine. (2022) 82:104136. doi: 10.1016/j.ebiom.2022.104136 35785620 PMC9256835

[70] Wang H Li M Zhang X He F Zhang S Zhao J . Impairment of peripheral Vdelta2 T cells in human cystic echinococcosis. Exp Parasitol. (2017) 174:17–24. doi: 10.1016/j.exppara.2017.01.005 28153802

[71] Jiang S Li H Zhang L . Generic Diagramming Platform (GDP): a comprehensive database of high-quality biomedical graphics. Nucleic Acids Res. (2025) 53:D1670–6. doi: 10.1093/nar/gkae973 39470721 PMC11701665

