# Supplementary material for: Gegen Qinlian decoction alleviates DSS-induced colitis in mice through coordinated modulation of gut microbiota, serum metabolome, and colonic γδT cell responses
Source: Front Immunol. 2026 Apr 2;17:1765637. doi: 10.3389/fimmu.2026.1765637 (PMC13083112; doi:10.3389/fimmu.2026.1765637)
Supplement: Supplementary file 1 [file Table1.docx]

Supplementary Material

Table S1 Disease activity scoring criteria

| Score | Weight Loss (%) | Stool Characteristics | Occult Blood in Stool |
| --- | --- | --- | --- |
| 0 | 0 | Normal | Normal (-) |
| 1 | 1-5 | Loose | Occult blood positive (+) |
| 2 | 5-10 | Loose | Slight bloody stool (++) |
| 3 | 10-15 | Diarrhetic | Gross bloody stool (+++) |
| 4 | ≥15 | Diarrhetic | Obvious bleeding (++++) |

|  |
| --- |

Table S2 Identified constitutes in GQD

| **No** | **time（min）** | **Ion mode** | **Formula** | **MW** | **Name** | **MS/MS data** | **Attribution** |
| --- | --- | --- | --- | --- | --- | --- | --- |
| 1 | 0.98 | [M-H]^-^ | C_4_H_6_O_5_ | 134.02 | Malic Acid | 133.0134;115.0027;71.0126 | Gancao |
| 2 | 1.18 | [M-H]^-^ | C_6_H_8_O_7_ | 192.03 | Citric acid | 191.0197;129.0181;111.0078;87.0076;57.0332 | Gegen;Huanglian;Gancao |
| 3 | 2.14 | [M-H]^-^ | C_15_H_20_O_10_ | 360.11 | (αR)-4-(β-D-glucopyranosyloxy)-α,3-dihydroxybenzenepropanoic acid | 359.0991;197.0452;179.0345;135.0444;123.0443;72.9919 | Huanglian |
| 4 | 2.45 | [M-H]^-^ | C_13_H_16_O_9_ | 316.08 | Protocatechuic acid-3-O-glucoside | 315.0729;152.0108;108.0206 | Gegen |
| 5 | 4.32 | [M-H]^-^ | C_8_H_14_O_6_ | 206.08 | Methyl quinate | 205.0717;143.0706;129.0549;115.0755;72.9919 | Gegen |
| 6 | 5.04 | [M-H]^-^ | C_10_H_10_O_5_ | 210.05 | (p-Hydroxybenzyl)malonic acid | 209.0459;165.0551;121.0649;119.0488;93.0334;59.0126 | Gancao |
| 7 | 5.11 | [M+H]^+^ | C_27_H_30_O_14_ | 578.16 | Puerarin 4'-O-glucoside | 579.1724;417.1189;399.1089;381.0980;351.0872;297.0766;267.0659 | Gegen |
| 8 | 5.66 | [M+H]^+^ | C_27_H_30_O_14_ | 578.16 | Daidzein-4',7-diglucoside | 579.1727;417.1194;255.0660 | Gegen |
| 9 | 5.83 | [M-H]^-^ | C_21_H_20_O_10_ | 432.11 | 3'-Hydroxypuerarin | 431.0995;311.0570;293.0465;283.0620;255.0672 | Gegen |
| 10 | 6.03 | [M-H]^-^ | C_17_H_20_O_9_ | 368.11 | 3-O-Feruloylquinic acid | 367.1040;193.0504;173.0452;149.0601;134.0365;117.0335 | Huanglian |
| 11 | 6.30 | [M-H]^-^ | C_26_H_28_O_14_ | 564.15 | 3'-Hydroxypuerarin 2′′-O-xyloside | 563.1416;341.0661;311.0569;293.0465;283.0620;255.0673 | Gegen |
| 12 | 6.60 | [M+H]^+^ | C_27_H_30_O_14_ | 578.16 | Puerarin 7'-O-glucoside | 579.1724;417.1193;399.1089;381.0984;351.0881;297.0768;267.0663 | Gegen |
| 13 | 6.64 | [M+H]^+^ | C_25_H_31_NO_10_ | 505.19 | Alangiside | 506.2038;344.1404;326.1386;190.0867 | Huanglian |
| 14 | 6.85 | [M-H]^-^ | C_21_H_20_O_9_ | 416.11 | Puerarin | 415.1045;295.0618;277.0512;267.0668;253.0495 | Gegen |
| 15 | 6.95 | [M+H]^+^ | C_26_H_28_O_13_ | 548.15 | 6′′-O-Apiofuranosylpuerarin | 549.1624;417.1192;399.1093;381.0979;351.0878;297.0767;267.0660 | Gegen |
| 16 | 7.01 | [M-H]^-^ | C_22_H_22_O_10_ | 446.12 | 3'-Methoxypuerarin | 445.1150;325.0723;310.0491;297.0773;282.0541 | Gegen |
| 17 | 7.15 | M^+^ | C_20_H_24_NO_4_^+^ | 342.17 | Magnoflorine | 342.1712;297.1132;282.0888;265.0866;237.0916;219.0797;191.0856 | Huanglian |
| 18 | 7.19 | [M+H]^+^ | C_27_H_30_O_14_ | 578.16 | 3'-Methoxymirificin | 579.1725;447.1296;429.1192;411.1087;381.0974;327.0874;297.0769 | Gegen |
| 19 | 7.41 | [M-H]^-^ | C_17_H_20_O_9_ | 368.11 | 4-O-Feruloylquinic acid | 367.1040;193.0557;173.0450;134.0365;93.0334 | Huanglian |
| 20 | 7.52 | [M+FA-H]^-^ | C_21_H_20_O_9_ | 416.11 | Daidzin | 461.1100;415.1045;253.0510 | Gegen |
| 21 | 7.85 | [M-H]^-^ | C_26_H_28_O_14_ | 564.15 | Schaftoside | 563.1415;503.1210;473.1104;443.0992;413.0903;395.0793;383.0782;353.0676 | Huangqin;Gancao |
| 22 | 7.92 | [M+H]^+^ | C_22_H_22_O_10_ | 446.12 | Calycosin-7-O-β-D-glucoside | 447.1300;285.0766;270.0533;253.0501;225.0558 | Gegen |
| 23 | 7.96 | [M-H]^-^ | C_21_H_20_O_10_ | 432.11 | Genistein 4′-O-glucoside | 431.0993;311.0569;269.0454 | Gegen |
| 24 | 8.03 | [M-H]^-^ | C_26_H_28_O_14_ | 564.15 | 6′′-O-Apiofuranosyl-3'-hydroxypuerarin | 563.1420;341.0667;311.0568;283.0618 | Gegen |
| 25 | 8.13 | [M+H]^+^ | C_26_H_33_NO_9_ | 503.22 | (13aS)-5,8,13,13a-Tetrahydro-3,9,10-trimethoxy-6H-dibenzo[a,g]quinolizin-2-yl β-D-glucopyranoside | 504.2252;342.1711;206.1183 | Huanglian |
| 26 | 8.19 | [M-H]^-^ | C_21_H_20_O_9_ | 416.11 | Neopuerarin | 415.1044;295.0617;277.0504;267.0668 | Gegen |
| 27 | 8.34 | [M-H]^-^ | C_27_H_30_O_14_ | 578.16 | Violanthin | 577.1576;487.1257;457.1135;325.0727;253.0509 | Gancao |
| 28 | 8.37 | [M-H]^-^ | C_21_H_20_O_9_ | 416.11 | Bayin | 415.1043;253.0508;252.0431;223.0388 | Gegen |
| 29 | 8.48 | [M-H]^-^ | C_26_H_28_O_13_ | 548.15 | Chrysin 6-C-arabinoside-8-C-glucoside | 547.1472;487.1278;457.1155;427.1038;367.0832;337.0727;309.0774 | Huangqin |
| 30 | 8.59 | [M-H]^-^ | C_21_H_22_O_9_ | 418.13 | Liquiritin | 417.1199;255.0667;135.0080;119.0492;91.0177 | Gancao |
| 31 | 8.79 | [M+FA-H]^-^ | C_21_H_20_O_10_ | 432.11 | Genistin | 477.1046;431.0993;311.0555;269.0462 | Gegen |
| 32 | 8.96 | [M+H]^+^ | C_26_H_28_O_13_ | 548.15 | Puerarin apioside | 549.1624;417.0985;399.1053;381.0983;321.0768 | Gegen |
| 33 | 8.98 | M^+^ | C_19_H_16_NO_4_^+^ | 322.11 | Groenlandicine | 322.1086;307.0849;294.1136;279.0901 | Huanglian |
| 34 | 9.01 | [M-H]^-^ | C_26_H_28_O_13_ | 548.15 | Chrysin 6-C-glucoside-8-C-arabinoside | 547.1473;457.1157;427.1041;367.0834;337.0728 | Huangqin |
| 35 | 9.15 | M^+^ | C_19_H_18_NO_4_^+^ | 324.12 | Demethyleneberberine | 324.1241;309.1008;294.0775;280.0977;266.0809 | Huanglian |
| 36 | 9.29 | [M+H]^+^ | C_27_H_30_O_13_ | 562.17 | Formononetin 8-C-[xylosyl(1→6)]-glucoside | 563.1772;431.1352;395.1146;311.0924;281.0817 | Gegen |
| 37 | 9.89 | [M+H]^+^ | C_23_H_22_O_10_ | 458.12 | 6''-O-Acetyldaidzin | 459.1310;255.0659;227.0702;199.0773 | Gegen |
| 38 | 9.98 | M^+^ | C_20_H_18_NO_4_^+^ | 336.12 | Epiberberine | 336.1242;320.0931;292.0980 | Huanglian |
| 39 | 10.03 | M^+^ | C_19_H_14_NO_4_^+^ | 320.09 | Coptisine | 320.0929;292.0977;277.0745;262.0883;234.0924 | Huanglian |
| 40 | 10.28 | [M-H]^-^ | C_26_H_30_O_13_ | 550.17 | Liquiritin apioside | 549.1627;255.0667;135.0080;119.0493 | Gancao |
| 41 | 10.48 | [M-H]^-^ | C_26_H_30_O_13_ | 550.17 | Isoliquiritin apioside | 549.1626;255.0668;135.0079;119.0492 | Gancao |
| 42 | 10.51 | M^+^ | C_20_H_20_NO_4_^+^ | 338.14 | Columbamine | 338.1400;322.1081;307.0851;294.1129;279.0902 | Huanglian |
| 43 | 10.56 | [M-H]^-^ | C_21_H_22_O_9_ | 418.13 | Isoliquiritin | 417.1201;255.0667;148.0159;135.0080;91.0177 | Gancao |
| 44 | 10.80 | [M+H]^+^ | C_22_H_22_O_9_ | 430.13 | Formononetin glucoside | 431.1346;269.0817;254.0574;213.0914 | Gegen;Gancao |
| 45 | 10.87 | [M+H]^+^ | C_15_H_10_O_4_ | 254.06 | Daidzein | 255.0659;237.0560 | Gegen |
| 46 | 11.07 | M^+^ | C_21_H_22_NO_4_^+^ | 352.15 | Palmatine | 352.1557;336.1243;320.1289;308.1293;292.0983 | Huanglian |
| 47 | 11.12 | [M-H]^-^ | C_15_H_12_O_4_ | 256.07 | Liquiritigenin | 255.0667;135.0080;119.0493;91.0180 | Gancao |
| 48 | 11.30 | [M-H]^-^ | C_21_H_20_O_11_ | 448.1 | Dihydrobaicalin | 447.0943;271.0619;253.0513;243.0665;225.0551;175.0240;152.0111 | Huangqin |
| 49 | 11.36 | M^+^ | C_20_H_18_NO_4_^+^ | 336.12 | Berberine | 336.1244;320.0927;306.0773;292.0985;278.0836 | Huanglian |
| 50 | 11.44 | [M+H]^+^ | C_21_H_18_O_11_ | 446.08 | Baicalin | 447.0939;429.7724;271.0610 | Huangqin |
| 51 | 11.59 | [M-H]^-^ | C_22_H_20_O_12_ | 476.1 | Diosmetin 7-O-glucuronide | 475.0891;299.0568;284.0333;228.0425;200.0471;175.0245;113.0235 | Huangqin |
| 52 | 11.76 | [M-H]^-^ | C_22_H_20_O_11_ | 460.1 | Wogonoside | 459.0948;283.0620;268.0385;175.0245;113.0235;85.0284 | Huangqin |
| 53 | 11.87 | [M-H]^-^ | C_21_H_18_O_10_ | 430.09 | Chrysin 7-O-glucuronide | 429.0833;253.0510;175.0244;113.0235 | Huangqin |
| 54 | 11.99 | [M-H]^-^ | C_22_H_20_O_12_ | 476.1 | 5,7,2'-Trihydroxy-6-methoxyflavone 7-O-glucuronide | 475.0892;299.0566;284.0331 | Huangqin |
| 55 | 12.29 | [M-H]^-^ | C_22_H_20_O_11_ | 460.1 | Oroxylin A 7-O-glucuronide | 459.0948;283.0618;268.0381;113.0233;85.0282 | Huangqin |
| 56 | 12.70 | [M-H]^-^ | C_21_H_18_O_11_ | 446.08 | Norwogonin 7-O-glucuronide | 445.0782;269.0461;241.0520;225.0560;197.0609;113.0233 | Huangqin |
| 57 | 12.91 | [M-H]^-^ | C_21_H_18_O_11_ | 446.08 | Norwogonin 8-O-glucuronide | 445.0778;269.0461;225.0553;197.0607 | Huangqin |
| 58 | 13.16 | [M-H]^-^ | C_44_H_64_O_18_ | 880.41 | 22β-acetoxyl-glycyrrhizin | 879.4041;351.0578;193.0352 | Gancao |
| 59 | 13.73 | [M-H]^-^ | C_15_H_10_O_5_ | 270.05 | Baicalein | 269.0462;251.0352;241.0505;223.0304;169.0655 | Huangqin |
| 60 | 13.94 | [M-H]^-^ | C_15_H_12_O_4_ | 256.07 | Isoliquiritigenin | 255.0667;135.0080;119.0493 | Gancao |
| 61 | 14.24 | [M-H]^-^ | C_42_H_62_O_17_ | 838.4 | Licorice saponin G2 | 837.3935;775.3864;661.3659;351.0577;289.0566;193.0351;175.0242 | Gancao |
| 62 | 14.31 | [M-H]^-^ | C_16_H_12_O_4_ | 268.07 | Formononetin | 267.0667;252.0432;223.0404 | Gegen;Gancao |
| 63 | 14.37 | [M-H]^-^ | C_42_H_60_O_16_ | 820.39 | Licoricesaponin E2 | 819.3832;351.0579;193.0351 | Gancao |
| 64 | 14.62 | [M-H]^-^ | C_42_H_62_O_17_ | 838.4 | Uralsaponin U | 837.3934;775.3927;485.3274;351.0576;289.0572;193.0351;175.0243 | Gancao |
| 65 | 14.67 | [M-H]^-^ | C_44_H_64_O_17_ | 864.41 | 22-β-acetoxyl-glycyrrhaldehyde | 863.4092;351.0577;193.0350;175.0242 | Gancao |
| 66 | 14.96 | [M-H]^-^ | C_42_H_62_O_16_ | 822.4 | Glycyrrhizic acid | 821.3984;759.4011;645.3679;351.0576;289.0583;235.0465;193.0351 | Gancao |
| 67 | 15.65 | [M+H]^+^ | C_16_H_12_O_5_ | 284.07 | Wogonin | 285.0766;270.0530 | Huangqin |
| 68 | 15.84 | [M-H]^-^ | C_42_H_62_O_16_ | 822.4 | Uralsaponin B | 821.3986;645.3694;351.0577;193.0352;175.0245;113.0234 | Gancao |
| 69 | 16.11 | [M-H]^-^ | C_19_H_18_O_8_ | 374.1 | Skullcapflavone II | 373.0935;358.0704;343.0466;328.0230;300.0275;285.0061;257.0087;169.0136 | Huangqin |
| 70 | 16.21 | [M+H]^+^ | C_16_H_12_O_5_ | 284.07 | Oroxylin A | 285.0766;270.0530 | Huangqin |
| 71 | 16.80 | [M-H]^-^ | C_18_H_16_O_7_ | 344.09 | Eupatilin | 343.0829;328.0595;313.0360;298.0124;270.0178;242.0226 | Gancao |

|  |
| --- |

Table S3 Significant Difference Metabolites between Con and DSS

| **NO** | **Compound** | **m/z** | **RT** | **FC** | **log2(FC)** | **pvalue** | **Levels** |
| --- | --- | --- | --- | --- | --- | --- | --- |
| 1 | Xanthine | 151.0260 | 207 | 11.6410 | 3.5412 | 0.0117 | up |
| 2 | Asiaticoside | 957.4861 | 192 | 4.5075 | 2.1723 | 0.0044 | up |
| 3 | 20-HDoHE | 327.2304 | 251 | 4.1464 | 2.0519 | 0.0015 | up |
| 4 | Glu_Val_Asn | 361.1712 | 290 | 2.9983 | 1.5841 | 0.0288 | up |
| 5 | Pantothenate | 218.1032 | 184 | 2.9880 | 1.5792 | 0.0009 | up |
| 6 | 2-Acetyl-8-hydroxy-3-methyl-1-naphthyl-D-glucopyranoside | 379.1388 | 421 | 2.8994 | 1.5358 | 0.0452 | up |
| 7 | Glu_Asp_Lys_Glu | 518.2086 | 688 | 2.8504 | 1.5111 | 0.0129 | up |
| 8 | CP_47,497-C8-homolog_C-8-hydroxy_metabolite | 347.258 | 105 | 2.8125 | 1.4918 | 0.0002 | up |
| 9 | 2,3-Dihydroxybenzoic_acid | 153.0184 | 119 | 2.7101 | 1.4383 | 0.0184 | up |
| 10 | Aspartic_acid | 134.0436 | 291 | 2.6003 | 1.3787 | 0.0164 | up |
| 11 | FAD/Flavin_adenine_dinucleotide | 784.1498 | 696 | 2.4429 | 1.2886 | 0.0006 | up |
| 12 | Xanthosine | 283.0681 | 257 | 2.4199 | 1.2750 | 0.0013 | up |
| 13 | 12(S),20-DiHETE | 359.2179 | 329 | 2.3964 | 1.2609 | 0.0320 | up |
| 14 | Leu_Ile | 245.192 | 324 | 2.2907 | 1.1958 | 0.0283 | up |
| 15 | 12(S)-HETE | 319.227 | 106 | 2.2615 | 1.1773 | 0.0007 | up |
| 16 | 3'-O-methylguanosine | 298.2731 | 239 | 2.1486 | 1.1034 | 0.0456 | up |
| 17 | Dodecanoylcarnitine | 344.2789 | 401 | 2.1071 | 1.0753 | 0.0229 | up |
| 18 | 3-(4-Hydroxyphenyl)lactic_acid | 181.0506 | 181 | 2.0870 | 1.0614 | 0.0000 | up |
| 19 | Prostaglandin_A1 | 335.2208 | 321 | 2.0467 | 1.0333 | 0.0195 | up |
| 20 | 15-deoxy-12,14-PGD2 | 333.2051 | 110 | 2.0438 | 1.0313 | 0.0044 | up |
| 21 | DL-2-Methylglutamic_acid | 160.0611 | 557 | 2.0388 | 1.0277 | 0.0006 | up |
| 22 | 1-Hexadecyl-2-(9Z-octadecenoyl)-sn-glycero-3-phosphoethanolamine | 702.5344 | 100 | 2.0165 | 1.0119 | 0.0190 | up |
| 23 | 4-Hydroxy-6-methylpyran-2-one | 127.0379 | 30 | 0.4923 | -1.0225 | 0.0072 | down |
| 24 | 2,4-Dihydroxybenzoic_acid | 155.1291 | 528 | 0.4896 | -1.0302 | 0.0127 | down |
| 25 | Estazolam | 295.0681 | 580 | 0.4870 | -1.0379 | 0.0420 | down |

Continued table S3 Significant Difference Metabolites between Con and DSS

| **NO** | **Compound** | **m/z** | **RT** | **FC** | **log2(FC)** | **pvalue** | **Levels** |
| --- | --- | --- | --- | --- | --- | --- | --- |
| 26 | 7,8-Dihydroneopterin | 254.2199 | 106 | 0.4792 | -1.0612 | 0.0019 | down |
| 27 | DL-Phenylalanine | 166.0858 | 298 | 0.4317 | -1.2120 | 0.0190 | down |
| 28 | L-Ornithine | 133.0962 | 578 | 0.3776 | -1.4051 | 0.0320 | down |
| 29 | Himbacine | 346.2733 | 259 | 0.3377 | -1.5661 | 0.0049 | down |
| 30 | Glyoxylate | 73.0292 | 178 | 0.3237 | -1.6273 | 0.0067 | down |
| 31 | Lys-Val | 246.1804 | 519 | 0.2936 | -1.7683 | 0.0011 | down |
| 32 | Succinic_acid | 117.0190 | 178 | 0.2907 | -1.7824 | 0.0055 | down |
| 33 | Sebacic_acid | 201.1128 | 117 | 0.2445 | -2.0318 | 0.0000 | down |
| 34 | Sorbitol_6-phosphate | 261.0424 | 116 | 0.2034 | -2.2974 | 0.0008 | down |
| 35 | Ala-Gly-Asp-Val | 359.1557 | 612 | 0.1564 | -2.6766 | 0.0002 | down |
| 36 | LysoPE(18:0/0:0) | 437.2620 | 120 | 0.1501 | -2.7361 | 0.0024 | down |
| 37 | Trimethylamine N-oxide | 76.0750 | 368 | 0.0597 | -4.0662 | 0.0006 | down |

Table S4 Significant Difference Metabolites between DSS and 5-ASA

| **NO** | **Compound** | **m/z** | **RT** | **FC** | **log2(FC)** | **pvalue** | **Levels** |
| --- | --- | --- | --- | --- | --- | --- | --- |
| 1 | Sebacic_acid | 201.1128 | 117 | 3.2268 | 1.6901 | 0.0001 | up |
| 2 | Lys-Val | 246.1804 | 519 | 5.0546 | 2.3376 | 0.0001 | up |
| 3 | DL-Phenylalanine | 164.0715 | 267 | 4.1573 | 2.0556 | 0.0011 | up |
| 4 | Ala-Gly-Asp-Val | 359.1557 | 612 | 2.9797 | 1.5752 | 0.0011 | up |
| 5 | Hydroxybutyrate | 137.0448 | 232 | 3.1926 | 1.6747 | 0.0012 | up |
| 6 | Succinic_acid | 117.0190 | 178 | 5.3391 | 2.4166 | 0.0016 | up |
| 7 | Indoxylsulfuric_acid | 212.0020 | 120 | 2.7633 | 1.4664 | 0.0020 | up |
| 8 | LysoPE(18:0/0:0) | 437.2620 | 120 | 6.7404 | 2.7528 | 0.0023 | up |
| 9 | Himbacine | 346.2733 | 259 | 4.4646 | 2.1585 | 0.0030 | up |
| 10 | 1-Palmitoyl-2-linoleoyl_PE | 716.5199 | 370 | 3.5326 | 1.8207 | 0.0033 | up |
| 11 | 10-Pyrene-PC | 850.5478 | 486 | 2.9820 | 1.5763 | 0.0035 | up |
| 12 | alpha-Ketoglutaric_acid | 145.0141 | 260 | 2.0828 | 1.0585 | 0.0036 | up |
| 13 | Xanthine | 151.0260 | 207 | 0.0593 | -4.0756 | 0.0039 | down |
| 14 | 7,8-Dihydroneopterin | 254.2199 | 106 | 2.1404 | 1.0979 | 0.0040 | up |
| 15 | L-Ornithine | 133.0962 | 578 | 4.8716 | 2.2844 | 0.0059 | up |
| 16 | Prostaglandin_A1 | 335.2208 | 321 | 0.4452 | -1.1675 | 0.0079 | down |
| 17 | 1-Methyl-L-histidine | 170.0914 | 433 | 0.4382 | -1.1902 | 0.0088 | down |
| 18 | Sorbitol_6-phosphate | 261.0424 | 116 | 2.8876 | 1.5299 | 0.0097 | up |
| 19 | 1-Hexadecanoyl-sn-glycero-3-phosphoethanolamine | 454.2912 | 410 | 2.3323 | 1.2218 | 0.0100 | up |
| 20 | 6-keto_PGE1 | 367.2101 | 115 | 0.4871 | -1.0376 | 0.0102 | down |
| 21 | Glyoxylate | 73.0292 | 178 | 2.3064 | 1.2057 | 0.0152 | up |
| 22 | Choline | 104.1068 | 446 | 2.3832 | 1.2529 | 0.0183 | up |
| 23 | 1,2-Dilinoleoyl-sn-glycero-3-phosphocholine | 782.5671 | 413 | 2.8924 | 1.5323 | 0.0194 | up |
| 24 | Gamma-Glu-Leu | 259.1295 | 405 | 2.1093 | 1.0768 | 0.0200 | up |
| 25 | Estazolam | 295.0681 | 580 | 2.1792 | 1.1238 | 0.0224 | up |
| 26 | Trimethylamine N-oxide | 76.0750 | 368 | 3.8453 | 1.9431 | 0.0357 | up |
| 27 | Malonic_acid | 103.0032 | 180 | 0.4060 | -1.3003 | 0.0420 | down |
| 28 | 12(R)-HETE | 303.2308 | 285 | 0.2720 | -1.8782 | 0.0462 | down |
| 29 | Diisodecyl_phthalate | 447.3452 | 322 | 3.9049 | 1.9653 | 0.0498 | up |

Table S5 Significant Difference Metabolites between DSS and GQD

| **NO** | **Compound** | **m/z** | **RT** | **FC** | **log2(FC)** | **pvalue** | **Levels** |
| --- | --- | --- | --- | --- | --- | --- | --- |
| 1 | 2-Oleoyl-1-palmitoyl-sn-glycero-3-phosphocholine | 760.5811 | 686 | 96.7980 | 6.5969 | 0.0020 | up |
| 2 | 1-Stearoyl-2-linoleoyl-sn-glycero-3-phosphocholine | 786.5979 | 517 | 83.8490 | 6.3897 | 0.0053 | up |
| 3 | 1-Palmitoyl-2-linoleoyl-sn-glycero-3-phosphocholine | 758.5683 | 399 | 73.6880 | 6.2034 | 0.0002 | up |
| 4 | 1-Palmitoyl-2-linoleoyl_PE | 716.5199 | 370 | 71.0660 | 6.1511 | 0.0000 | up |
| 5 | 1-Hexadecyl-2-(5Z,8Z,11Z,14Z-eicosatetraenoyl)-sn-glycero-3-phosphocholine | 768.5882 | 341 | 69.3540 | 6.1159 | 0.0018 | up |
| 6 | 10-Pyrene-PC | 850.5478 | 486 | 54.6130 | 5.7712 | 0.0001 | up |
| 7 | 2-Methylguanosine | 298.2736 | 214 | 49.0840 | 5.6172 | 0.0257 | up |
| 8 | Choline | 104.1068 | 446 | 46.8940 | 5.5513 | 0.0000 | up |
| 9 | 1,2-Dilinoleoyl-sn-glycero-3-phosphocholine | 782.5671 | 413 | 43.8010 | 5.4529 | 0.0004 | up |
| 10 | PE(16:1(5Z)/16:1(5Z)) | 688.4881 | 332 | 42.7570 | 5.4181 | 0.0006 | up |
| 11 | 1,2-Dilinoleoyl-sn-glycero-3-phosphoethanolamine | 740.5195 | 376 | 32.4440 | 5.0199 | 0.0002 | up |
| 12 | Eicosapentaenoyl_PAF_C-16 | 766.5719 | 304 | 31.6210 | 4.9828 | 0.0035 | up |
| 13 | 1-Methylhistamine | 126.1014 | 494 | 27.7570 | 4.7948 | 0.0430 | up |
| 14 | Diisodecyl_phthalate | 447.3452 | 322 | 24.8310 | 4.6341 | 0.0175 | up |
| 15 | Hydroxybutyrate | 105.1096 | 446 | 23.0720 | 4.5281 | 0.0000 | up |
| 16 | 2-Docosahexaenoyl-1-palmitoyl-sn-glycero-3-phosphoethanolamine | 764.5225 | 319 | 21.5110 | 4.4270 | 0.0000 | up |
| 17 | 1-Heptadecanoyl-sn-glycero-3-phosphocholine | 510.3533 | 220 | 20.1310 | 4.3313 | 0.0299 | up |
| 18 | 1-Octadecanoyl-sn-glycero-3-phosphoethanolamine | 482.3225 | 213 | 19.2560 | 4.2672 | 0.0247 | up |
| 19 | 17-HDoHE | 327.2315 | 208 | 18.4810 | 4.2079 | 0.0350 | up |
| 20 | Sphinganine | 302.3043 | 305 | 14.3910 | 3.8471 | 0.0053 | up |

Continued table S5 Significant Difference Metabolites between DSS and GQD

| **NO** | **Compound** | **m/z** | **RT** | **FC** | **log2(FC)** | **pvalue** | **Levels** |
| --- | --- | --- | --- | --- | --- | --- | --- |
| 21 | Butanoyl_PAF | 552.4000 | 299 | 13.4580 | 3.7504 | 0.0016 | up |
| 22 | PC(O-16:0/0:0) | 482.3577 | 288 | 11.3040 | 3.4987 | 0.0079 | up |
| 23 | Glycerol_1-stearate | 359.3143 | 278 | 10.8040 | 3.4334 | 0.0041 | up |
| 24 | Fexofenadine | 502.2911 | 214 | 9.3714 | 3.2283 | 0.0407 | up |
| 25 | Methylcarbamyl_PAF_C-16 | 539.3884 | 268 | 8.0702 | 3.0126 | 0.0036 | up |
| 26 | 12(R)-HETE | 303.2308 | 285 | 7.3590 | 2.8795 | 0.0130 | up |
| 27 | 17-phenyl_trinor_Prostaglandin_F2_serinol_amide | 462.2833 | 287 | 7.3332 | 2.8744 | 0.0037 | up |
| 28 | L-Ornithine | 133.0962 | 578 | 7.0078 | 2.8090 | 0.0038 | up |
| 29 | Leu_Leu_Leu_Ala_Ala | 500.3486 | 271 | 6.6868 | 2.7413 | 0.0016 | up |
| 30 | L-Pipecolic_acid | 130.0853 | 557 | 5.8473 | 2.5478 | 0.0452 | up |
| 31 | Himbacine | 346.2733 | 259 | 5.5018 | 2.4599 | 0.0112 | up |
| 32 | Lys-Val | 246.1804 | 519 | 5.4013 | 2.4333 | 0.0001 | up |
| 33 | Trimethylamine N-oxide | 76.0750 | 368 | 5.3494 | 2.4194 | 0.0034 | up |
| 34 | LysoPE(18:0/0:0) | 437.2620 | 120 | 5.3024 | 2.4067 | 0.0034 | up |
| 35 | 1,2-Ditetradecanoyl-sn-glycero-3-phosphocholine | 678.4995 | 397 | 4.5340 | 2.1808 | 0.0012 | up |
| 36 | DL-Phenylalanine | 166.0858 | 298 | 3.5869 | 1.8427 | 0.0275 | up |
| 37 | Sebacic_acid | 201.1128 | 117 | 3.0146 | 1.5920 | 0.0004 | up |
| 38 | Monensin_sodium_salt | 693.8664 | 475 | 2.9104 | 1.5412 | 0.0056 | up |
| 39 | Tyr_Val_Tyr_Lys_Val | 671.3746 | 473 | 2.8822 | 1.5272 | 0.0002 | up |
| 40 | Sorbitol_6-phosphate | 261.0424 | 116 | 2.6574 | 1.4100 | 0.0085 | up |
| 41 | Estazolam | 295.0681 | 580 | 2.6187 | 1.3888 | 0.0113 | up |
| 42 | Zebularine | 227.0659 | 395 | 2.6142 | 1.3864 | 0.0001 | up |
| 43 | Glyoxylate | 73.0292 | 178 | 2.5793 | 1.3670 | 0.0134 | up |
| 44 | Ala-Gly-Asp-Val | 359.1557 | 612 | 2.5095 | 1.3274 | 0.0032 | up |
| 45 | Indoxylsulfuric_acid | 212.0020 | 120 | 2.3082 | 1.2068 | 0.0038 | up |
| 46 | Succinic_acid | 117.0190 | 178 | 2.2700 | 1.1827 | 0.0293 | up |
| 47 | D-Glucose | 179.0562 | 396 | 2.1572 | 1.1092 | 0.0021 | up |
| 48 | b-D-Galactopyranose | 179.0561 | 428 | 2.1521 | 1.1057 | 0.0089 | up |
| 49 | FAD/Flavin_adenine_dinucleotide | 784.1498 | 696 | 0.4930 | -1.0203 | 0.0345 | down |
| 50 | ADMA | 203.1495 | 624 | 0.4929 | -1.0207 | 0.0427 | down |
| 51 | 2,3-Dihydroxymandelic_acid | 153.0184 | 119 | 0.4592 | -1.1230 | 0.0086 | down |
| 52 | L-Leucine | 130.0875 | 279 | 0.4590 | -1.1236 | 0.0005 | down |
| 53 | 2-thio-PAF | 540.3472 | 489 | 0.4578 | -1.1272 | 0.0208 | down |

Continued table S5 Significant Difference Metabolites between DSS and GQD

| **NO** | **Compound** | **m/z** | **RT** | **FC** | **log2(FC)** | **pvalue** | **Levels** |
| --- | --- | --- | --- | --- | --- | --- | --- |
| 54 | Enalaprilat | 349.1820 | 332 | 0.4260 | -1.2311 | 0.0481 | down |
| 55 | L-beta-Homoproline | 130.0851 | 688 | 0.4218 | -1.2454 | 0.0051 | down |
| 56 | PGA1 | 335.2223 | 108 | 0.4170 | -1.2620 | 0.0005 | down |
| 57 | 1113,14-dihydro-15-keto_PGF2 | 353.2322 | 113 | 0.4155 | -1.2671 | 0.0000 | down |
| 58 | CP_47,497-C8-homolog_C-8-hydroxy_metabolite | 347.2580 | 105 | 0.3310 | -1.5950 | 0.0004 | down |
| 59 | 12(S)-HETE | 319.2270 | 106 | 0.3271 | -1.6123 | 0.0006 | down |
| 60 | Leu_Ile | 245.1920 | 324 | 0.1757 | -2.5087 | 0.0208 | down |
| 61 | Uric_acid | 167.0210 | 351 | 0.1667 | -2.5847 | 0.0300 | down |
| 62 | Phosphocholine | 184.0722 | 313 | 0.1513 | -2.7241 | 0.0257 | down |
| 63 | PGF2_1,9-lactone | 319.2249 | 329 | 0.1402 | -2.8344 | 0.0322 | down |
| 64 | 12(S),20-DiHETE | 359.2179 | 329 | 0.1356 | -2.8827 | 0.0147 | down |
| 65 | Prostaglandin_A1 | 335.2208 | 321 | 0.1240 | -3.0112 | 0.0394 | down |
| 66 | Glu_Val_Asn | 361.1712 | 290 | 0.1070 | -3.2250 | 0.0263 | down |
| 67 | Cystine | 239.0163 | 829 | 0.1035 | -3.2719 | 0.0251 | down |
| 68 | isocarboxazid | 232.1025 | 487 | 0.1021 | -3.2926 | 0.0100 | down |
| 69 | 1-hydroxypyrene | 219.0816 | 494 | 0.0981 | -3.3499 | 0.0131 | down |
| 70 | Histidine | 156.0758 | 379 | 0.0948 | -3.3982 | 0.0035 | down |
| 71 | Xanthine | 151.0260 | 207 | 0.0893 | -3.4845 | 0.0000 | down |
| 72 | Puerarin | 415.1024 | 175 | 0.0841 | -3.5725 | 0.0002 | down |
| 73 | Aspartic_acid | 134.0436 | 291 | 0.0786 | -3.6685 | 0.0294 | down |
| 74 | Thr_Gln_Leu_Lys | 489.3025 | 408 | 0.0450 | -4.4734 | 0.0364 | down |
| 75 | 3-O-Benzyl-16-O-tert-butoxycarbonyl-16-alpha-hydroxyestrone | 461.2712 | 405 | 0.0338 | -4.8870 | 0.0300 | down |
| 76 | Dodecanoylcarnitine | 344.2789 | 401 | 0.0320 | -4.9660 | 0.0497 | down |
| 77 | Leu_Ile_Asp_Arg | 516.3051 | 401 | 0.0302 | -5.0504 | 0.0403 | down |
| 78 | Palmitoyl-L-carnitine | 400.3414 | 406 | 0.0251 | -5.3186 | 0.0477 | down |
| 79 | 2-O-methyl_PAF_C-18 | 524.4021 | 473 | 0.0216 | -5.5336 | 0.0295 | down |

Table S6 Shared differential metabolites

| **NO** | **Compound** | **Formular** | **m/z** | **Compared to DSS** | | |
| --- | --- | --- | --- | --- | --- | --- |
|  |  |  |  | **Con** | **5-ASA** | **GQD** |
| 1 | LysoPE(18:0/0:0) | C_23_H_48_NO_7_P | 437.262 | down | down | down |
| 2 | Succinic acid | C_4_H_6_O_4_ | 117.019 | down | down | down |
| 3 | Lys-Val | C_11_H_23_N_3_O_3_ | 246.1804 | down | down | down |
| 4 | L-Ornithine | C_5_H_12_N_2_O_2_ | 133.0962 | down | down | down |
| 5 | Himbacine | C_22_H_35_NO_2_ | 346.2733 | down | down | down |
| 6 | DL-Phenylalanine | C_9_H_11_NO_2_ | 166.0858 | down | down | down |
| 7 | Trimethylamine N-oxide | C_3_H_9_NO | 76.075 | down | down | down |
| 8 | Sebacic acid | C_10_H_18_O_4_ | 201.1128 | down | down | down |
| 9 | Ala-Gly-Asp-Val | - | 359.1557 | down | down | down |
| 10 | Sorbitol-6-phosphate | C_6_H_15_O_9_P | 261.0424 | down | down | down |
| 11 | Glyoxylate | C_2_H_2_O_3_ | 73.0292 | down | down | down |
| 12 | Estazolam | C_16_H_11_ClN_4_ | 295.0681 | down | down | down |
| 13 | Prostaglandin A1 | C_20_H_30_O_4_ | 335.2208 | up | up | up |
| 14 | Xanthine | C_5_H_4_N_4_O_2_ | 151.026 | up | up | up |


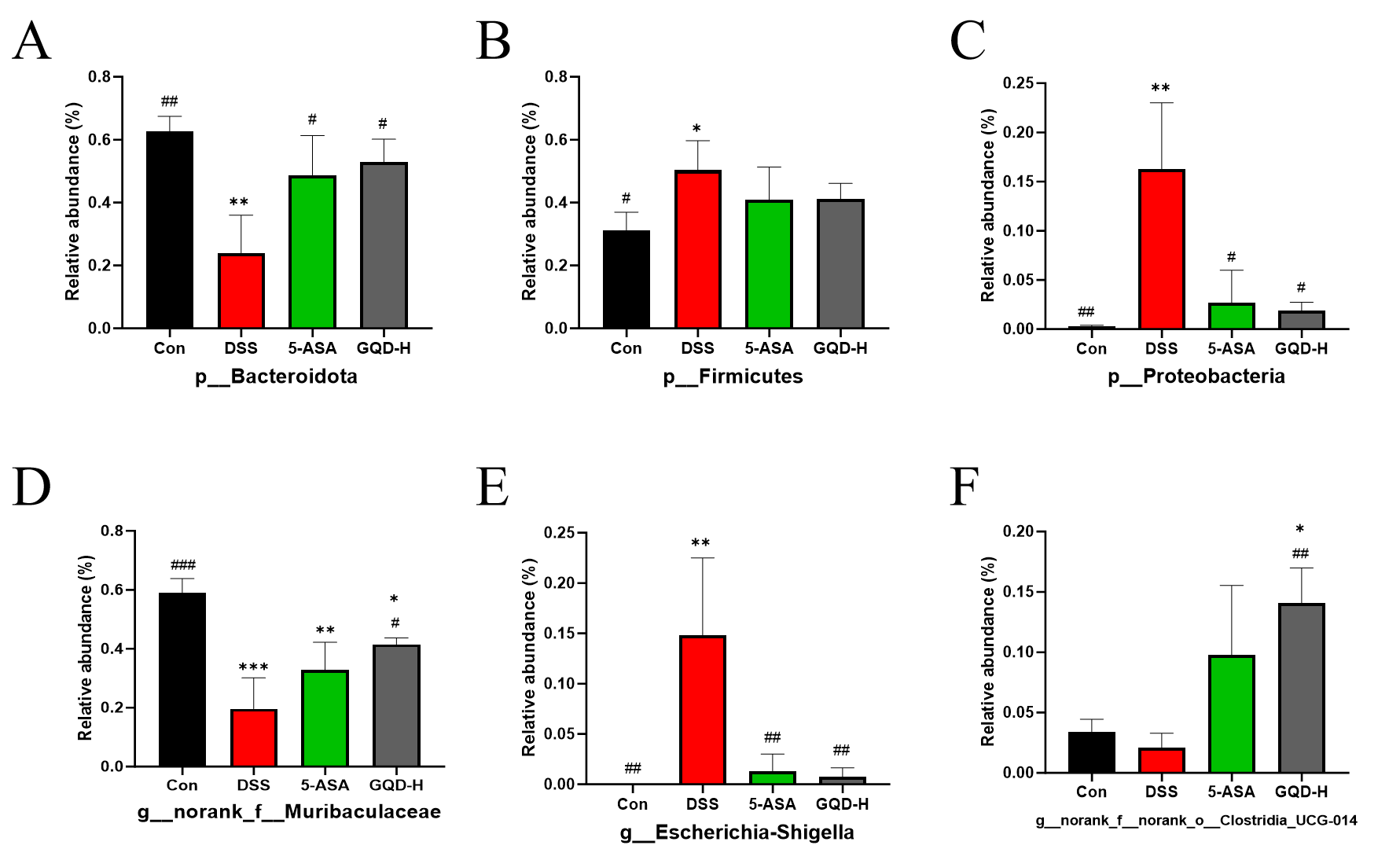


Fig S1 GQD ameliorates symptoms in UC mice.

1. Relative abundance of phylum Bacteroidota. (B) Relative abundance of phylum Firmicutes. (C) Relative abundance of phylum Proteobacteria. (D) Relative abundance of genus norank_f__Muribaculaceae. (E) Relative abundance of genus Escherichia-Shigella.(F) Relative abundance of genus norank_f__norank_o__Clostridia_UCG-014. Data are presented as mean ± SEM (n = 3 per group). Compared with the Con group: *P < 0.05, **P < 0.01. Compared with the DSS group: #P < 0.05, ##P < 0.01.


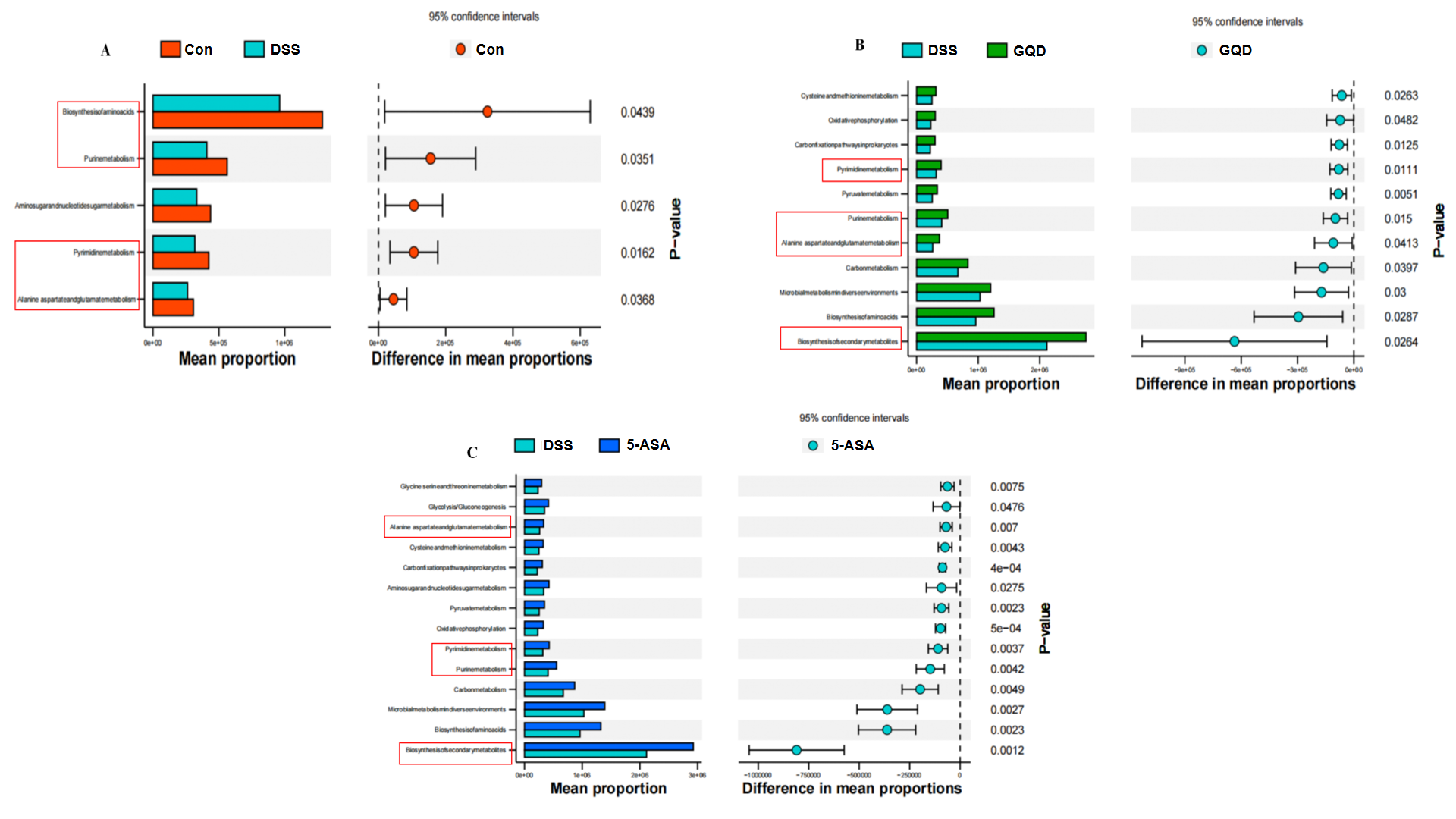


Fig S2 Picrust2 function prediction

1. Con vs DSS；(B) DSS vs GQD；(C) DSS vs 5-ASA

Fig S3 Relative intensity of Trimethylamine N-oxide in each group

Compared with the Con group: ***P < 0.001. Compared with the DSS group: ###P < 0.001.


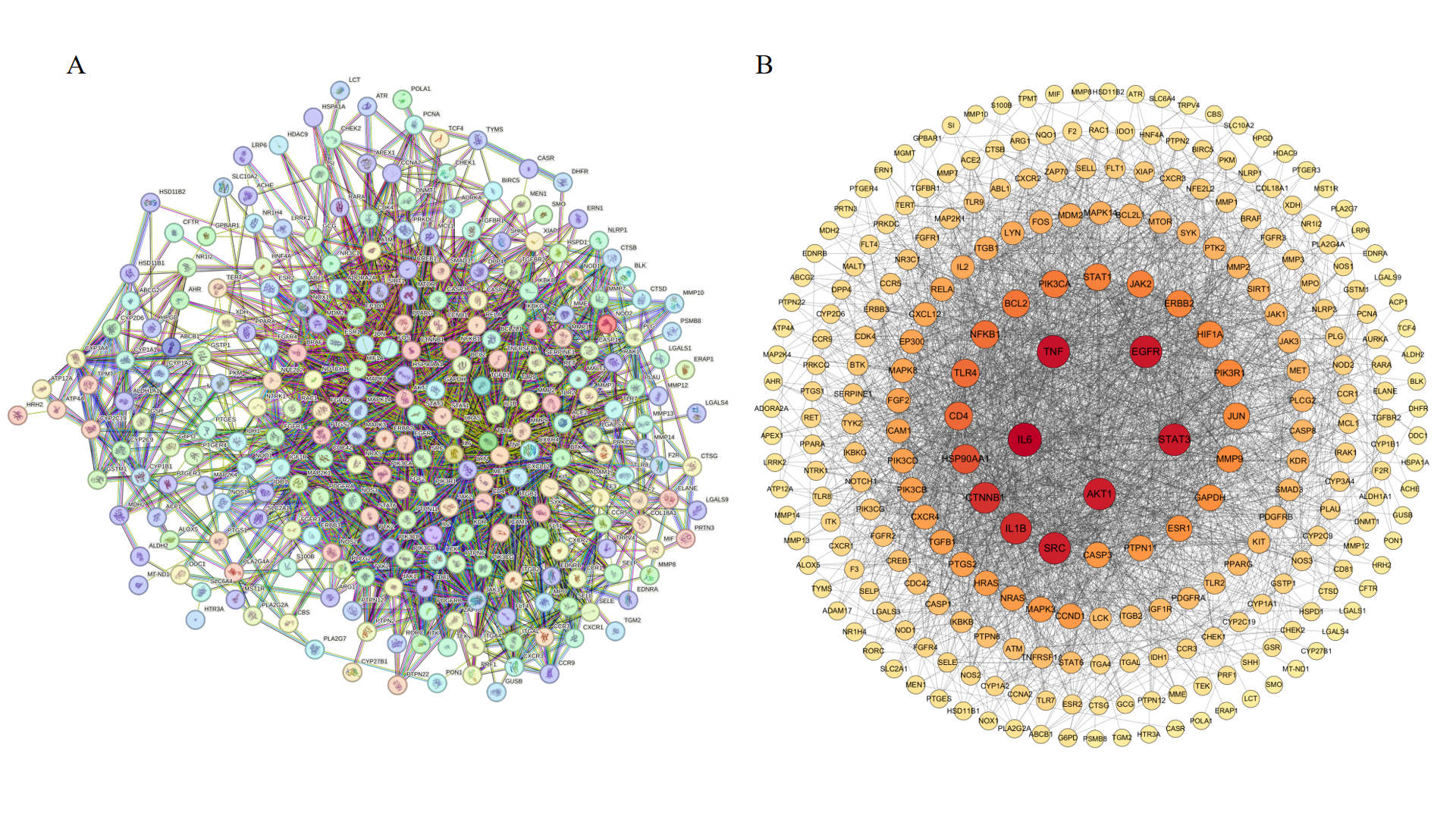


Fig S4 PPI Network Diagram for GQD Treatment of UC

(A) The PPI network includes 281 intersection genes; (E) Core Module Analysis of the PPI Network.
